# A complex systems perspective of news recommender systems: Guiding emergent outcomes with feedback models

**DOI:** 10.1371/journal.pone.0245096

**Published:** 2021-01-07

**Authors:** Shankar Prawesh, Balaji Padmanabhan

**Affiliations:** 1 Industrial and Management Engineering, IIT Kanpur, Kanpur, UP, India; 2 Muma College of Business, University of South Florida, Tampa, FL, United States of America; University of Pisa, ITALY

## Abstract

Algorithms are increasingly making decisions regarding what news articles should be shown to online users. In recent times, unhealthy outcomes from these systems have been highlighted including their vulnerability to amplifying small differences and offering less choice to readers. In this paper we present and study a new class of *feedback models* that exhibit a variety of self-organizing behaviors. In addition to showing important emergent properties, our model generalizes the popular “top-N news recommender systems” in a manner that provides media managers a mechanism to guide the emergent outcomes to mitigate potentially unhealthy outcomes driven by the self-organizing dynamics. We use complex adaptive systems framework to model the popularity evolution of news articles. In particular, we use agent-based simulation to model a reader’s behavior at the microscopic level and study the impact of various simulation hyperparameters on overall emergent phenomena. This simulation exercise enables us to show how the feedback model can be used as an alternative recommender to conventional top-N systems. Finally, we present a design framework for multi-objective evolutionary optimization that enables recommendation systems to co-evolve with the changing online news readership landscape.

## Introduction

There is growing concern about some undesirable aspects of user interaction with news recommender systems (from here on referred to as “NRS”). The overabundance of contents vying for limited attention of users creates a cannibalization effect in social media leading to a “winner take all” effect among news articles [1] where a few articles receive most of the viewership and reader engagement [2]. This can, however, create and lead to poorly informed societies. We have noticed numerous incidents exploiting these vulnerabilities to spread misinformation and fake news through social media [3, 4]. The speed with which information could be disseminated on social media creates also an opportunity to target recommendation engines for generating clickbait to increase popularity of the target articles [5].

In the context of recommender systems, Morik et al. [6] note that the naïve implementation of ranking algorithms leads to at least two undesirable outcomes that require careful consideration at the design stage. First, the ranking system induces a bias through the ranking because the clicks of an article are not consistent estimators of its average relevance (i.e. fraction of users that want to read the article). In fact, the articles which get a chance to be displayed in early iterations are ranked highly, could be easily found by users and they receive more clicks over time. This causes a “rich get richer” phenomenon where the clicks received by articles does not reflect their true relevance to users. Second, the ranking is an arbiter of how much exposure each item receives which directly influences user behavior and opinion [6]. An algorithm is fair if the exposure of an item is linked to its merit. For example, consider the Nth and (N+1)th article in a system that uses top-N recommendation. A top-N recommender sorts articles using a metric of interest (e.g. clicks) and selects N articles corresponding to the highest metric values for display. Assume that the difference in their clicks is miniscule (0 or 1) before the recommendation, i.e. they have similar merit. Using a naïve ranking algorithm, the Nth article will be selected for recommendation and over time it will receive disproportionately higher visibility. Here the relevance of both articles (N and (N+1)) is known, but the ranking policy is the source of unfairness.

How can such undesirable outcomes could be prevented? Previously researchers have used causal inference, algorithmic adjustments for click bias, and randomized interventions to address some of these challenges [6]. In this paper, we take a different approach and present a simple yet powerful mechanism using a complex systems perspective that can be used to guide emergent outcomes in a way that mitigates the undesirable outcomes. While our results and exposition are specific to NRS it is easy to see how our approach can generalize to other types of recommendations as well.

Specifically, we propose a solution motivated by complex systems ideas referred to as *feedback* based NRS (or “FNRS”) and discuss important properties of it using agent based model. An FNRS selects N articles for recommendation probabilistically using the feedback function (Algorithm 1). Feedback based systems provide a mechanism where the NRS can be *guided* to self-organize in specific *desirable* ways, for example, promoting the popular articles or new articles with low click counts, bringing diversity in recommendation etc. Thereby FNRS offers important flexibility for media managers in generating the recommendation list. While these ideas can also be used by social media platforms, in our work we primarily focus on online news platforms when presenting our ideas and discussing their impact. Feedback models have been extensively studied before [7–9] where the behavior of a complex system creates either positive or negative feedback that affects the future behavior of the system. Our adaptation of these models (Eq 3) to guide emergent behavior in news recommenders is one of the main contributions of this research.

In the feedback models proposed here the recommendation probability of an article with click count *c* is proportional to f(*c*) = c^γ^,γ ∈ ℝ. Increasing value of the exponent (γ > 1) creates positive feedback, and biases the system toward selection of the currently best fit (popular) articles, while crowding out less fit articles. γ ≤ 0, creates negative feedback in the system, that allows an implementer to drive traffic to long-tail articles or to design novelty in the recommendation. The most popular top-N recommender widely used today uses a hard cutoff after selecting the “best *N* articles” (the ones with the highest readership counts), hence this correspond to γ → ∞.

The feedback model offers a wide range of flexibility to introduce diversity in selection of articles to generate the recommendation list. Introducing diversity in recommendation is recognized as one of the important characteristics by practitioners, and in general accuracy and diversity both are considered important aspects of recommendation [10].

Modeling the news ecosystem as a Complex Adaptive System (CAS) [11–13] allows our feedback-based solution mechanism to generate a variety of article evolution scenarios by tuning the parameter γ. This, in and of itself, helps mitigate some of the unhealthy dynamics as we show in this paper. However, we build on this idea further and develop a framework that can be used to choose this parameter dynamically in a multi-objective setting that can help continuously guide emergent behavior of online readership as the landscape evolves (Algorithm 2).

Our work adopts a complex systems perspective, which is an appropriate framework to study the impact of algorithms on users due to the fact that this perspective explicitly models how users and other agents respond in the environment in which these algorithms are deployed [14]. Feedback mechanisms—a central theme of the model of recommendation studied in this paper—are a commonly observed phenomenon among self-organizing complex adaptive systems. A positive feedback mechanism can cause a butterfly effect [15] in which an entity grows super-linearly, eventually reaching a monopoly status by starting with some initial negligible advantage over the other members [8]. Models generating positive feedback are commonly used to model social sharing process. For example, to model diffusion amplification Yoo et al. (2019) [1] use self-excited Hawkes process which models complex contagion based on how many times a piece of content is shared. Each time a piece of content is shared, its intensity of sharing increases.

Preferential attachment based models also make a similar kind of contribution in terms of modeling feedback in networks. Barabasi et al. [16] in a classic paper first reported an unexpected high degree to self-organization in the large-scale properties of complex networks resulting in emergent behavior into scale-free states. While there is a lot of work in network models, much of this literature discusses different growth models [17] and the impact they have on emergent outcomes. While less common, in this literature too there are notions of unhealthy emergent scenarios and algorithmic interventions to mitigate. One example is the interesting work in [18] where they address how to “attack preferential attachment models” in the case of mobile peer to peer ad-hoc networks. In this application, they show how existing network routing algorithms still cause over-reliance on a few nodes that can lead to battery death and failures (since these are peer to peer networks) and propose alternative routing algorithms.

In addition to showing the value of feedback based models to control news recommender systems, in this paper we address the time-evolving aspect of the problem as well. This dynamic nature of the news ecosystem gives rise to a complex multi-objective optimization which poses characteristics of “wicked problems” [19, 20]. Wicked problems emerge from interaction between human and sociotechnical systems which has multiple objectives and stakeholders [21]. Wicked problems do not have an optimal solution, but algorithms could be developed to “tame” them [21]. In this context, we develop a framework for multi-objective optimization to design a dynamic FNRS. Recognizing that designing news recommendation poses a wicked problem to decision makers, we develop a framework for dynamic multi-objective news recommendation. We quantify two conflicting objectives: *accuracy-loss* and *distortion* (see Eqs 6–8). We say that the system generates distortion if it promotes articles in such a way, that the click-count share of popular articles increases, and the less popular articles decreases due to recommendation. This phenomenon is also known as rich-get-richer dynamics [6]. Whereas accuracy-loss is defined by considering the articles selected as recommendation using sorting algorithm that uses hard-cutoff as ground truth. Therefore, any other selection mechanism that leads to selection of a less popular article will incur accuracy loss. We use accuracy-loss and distortion for the multi-objective problem and present an evolutionary algorithm for the continuous update of the recommendation list where the system adapts to its environment using a combination of *exploration* and *exploitation* [12].

There are significant gaps in the recommender systems and CAS literature that our research addresses. First, our paper bridges the gap between NRS and CAS and opens the door for more work that can contribute to news recommender systems by using complex systems frameworks. Second, there is not much work in the news recommendation literature on how to *guide* emergent outcomes in terms of which articles get promoted and read. The motivating theories driving our idea suggest that desired emergent outcomes are possible by a process of exploration and exploitation. The new feedback-based model presented here, and the multi-objective framework are hence important contributions to the theory in both the recommender systems and the complex systems area, where this is a significant gap in the literature.

As is now customary, all the code developed in this work are released with the paper for future enhancements or research extensions.

## Modeling the news ecosystem as a complex adaptive system

We design an agent-based simulation of a typical news platform as a mechanism to study its emergent properties. From a methodological perspective agent-based modeling is a widely used mechanism for studying complex systems [22, 23]. Simulations are particularly suited for studying complex systems since they exhibit non-linear behavior and emergent properties unravel over time [24, 25]. Simulation also allows us to experiment with various parameters and *what-if* cases.

In news recommendation, social media and algorithmic decision-making fuel complexity by fostering dependence among human actors, technical artefacts, process, organizations and institutions [21]. In particular, in Information Systems (IS) literature, CAS perspective has been used to identify the enablers and inhibitors of agility and the emergent capability of agile teams in the context of agile software development [26]. They consider match coevolutionary rate, self-organization optimization and synchronization between exploration and exploitation as the core principles of CAS-grounded study of agile software. In the similar vein, we consider self-organization and exploration and exploitation as the integral part of complex systems driven design of news recommendation.

The major components–the environment, the agents in it, and the interaction in the system—are summarized in Table 1. The environment is the medium for agents to operate and interact with. This is the online news ecosystem, which is implemented in our simulation through a Web site similar to popular news sites including multiple pages corresponding to different categories of information. Agents include readers who constantly arrive and read articles as well as the media manager who helps determine the exact mechanism for the recommendation. The interaction between agents occurs through the most popular list which reflects the preference of all agents.

**Table 1:** Basic components of agent based simulation.

Concept	Description	Execution
Environment	Medium for agents to operate on and interact with.	• This is the online news ecosystem, implemented as a Website
		• Online news website’s structure (front page, related pages, how the categories are displayed) are done to mirror popular sites such as nytimes.com or cnn.com
		• Online news website’s lists that highlight articles to read in each category as well as the recommender systems box
		• Arrival of new articles and retiring of old articles
Agent	Individual actors or basic entities of actions	Readers
		• *Attribute* of readers–no. of articles read by them, reading probability
		• *Behavioral* rules of readers–read the recommended and recent articles with a higher probability. The focal agent adapts her behavior, based on “local information” provided to her through the recommended and the display lists
		Media-managers
		• Media-managers plays the role of policy intervention in the system. They use insights gained from the self-organizing property of FNRS (Eq 1) to steer the selection of articles in the recommended list. They update database with necessary information about each article and also update all Web pages as a new article arrives in the system
Interaction	Adaptive behavior of agents	• Interaction takes place among agents indirectly through most-popular list—which reflects the preference of other agents. The level of interaction among agents is determined by the probability with which a reader selects articles from the recommended list.

### Environment

**Fig 1:**
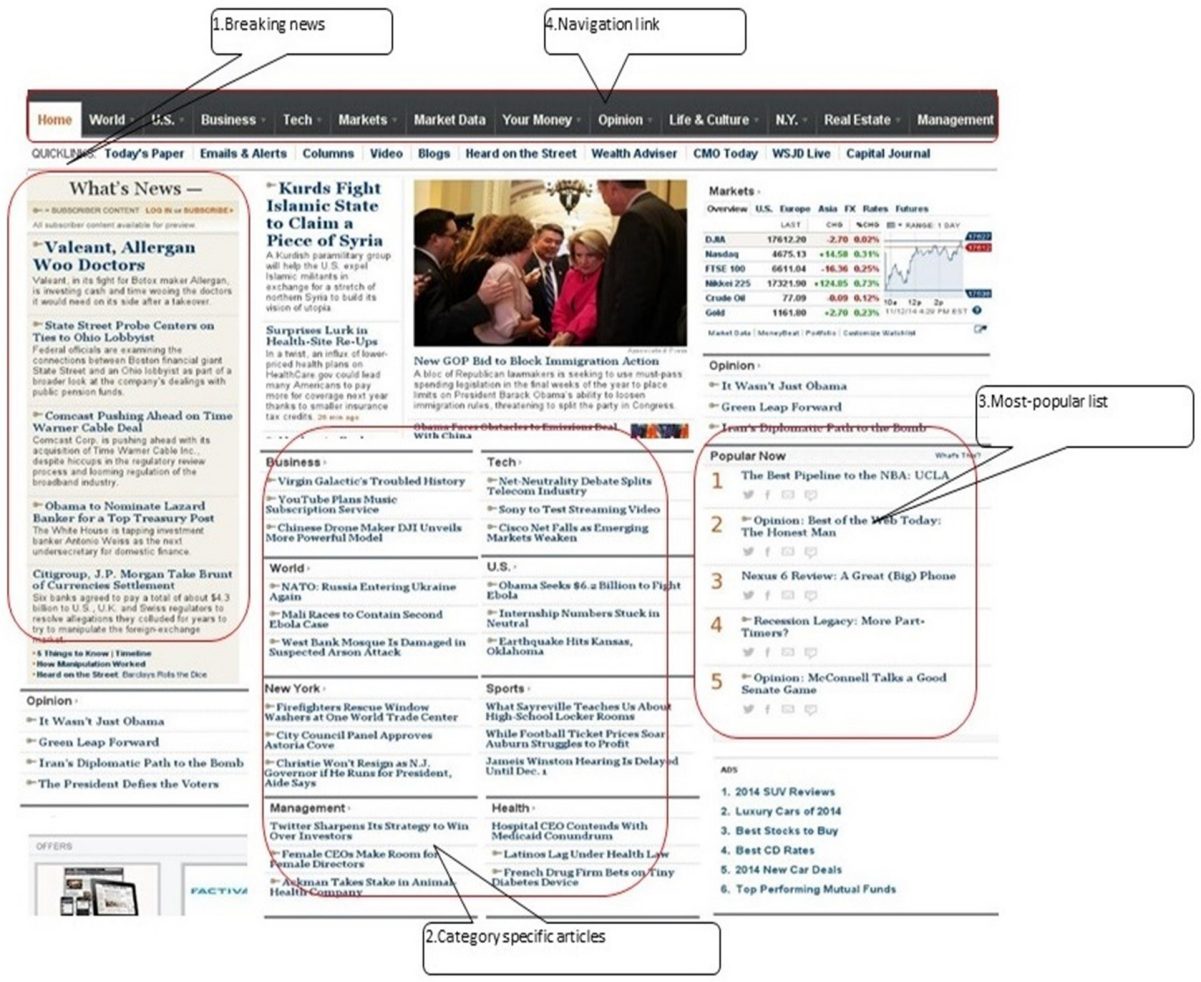
Common features available on the front page of a news website.

We implemented this exactly like a typical news site similar to Fig 1 below. Usually the front page consists of a section called Breaking News, which displays a pre-determined number of articles chronologically. Other important sections are: the chronological display of articles corresponding to each of the different *categories*, and the “most-popular” list. Commonly used news categories across different news websites are: Local News, World, Weather, Sports, Health, Business, Technology and Politics. The displayed articles in different categories are updated on arrival of a new article, whereas the most popular list of articles is updated as the reading activity progresses with arrival of readers.

The links to navigate through different categories are available on the top of a page (except at the front page). When the reader clicks on one of the links corresponding to a category, then ten most recent articles corresponding to the category are displayed. When a reader clicks any of the articles, the link takes her to the page which also displays a list of 10 most-popular articles. As a common practice, the most-popular or top-N list is usually displayed prominently on each Web page of most of the news sites.

Before the simulation starts, we generate several hundred news articles with an even distribution across categories with unique ids (UID) for the articles. Each article was assigned a random initial count, drawn from a random variable that follows Zipf’s distribution [27]. The Zipf distribution is a discrete distribution defined over a finite set *k* ∈ (1…*n*) with probability mass function,

$$p(k)=\frac{\frac{1}{k^{s}}}{\sum_{k=1}^{n}\left(\frac{1}{k^{s}}\right)};s>0$$ (1)

We use *n* = 1000 in Eq 1 to generate the initial click counts of articles. The arrival of new articles in the system was simulated using a Poisson process with arrival rate *ρ*. Upon arrival of an article it is randomly assigned to a category and its timestamp is noted.

One of the weaknesses shown previously [2] in top-N systems is the disproportional drop in recognition to the (N+1)th article in a list, unknowingly penalized for “not making the cutoff”. To analyze this in a greater detail, we keep the initial reader count difference between 10^th^ and 11^th^ articles to be just one to resemble “*tiny initiating events*” [28] in CAS.

### Agents

Individual agents may exhibit heterogenous behavior in real life. But even a simple representation of their behavior in model can help us generate the phenomena observed at the aggregate level. For example, consider the Schelling’s segregation model [29]–although not related to recommendation, it does help us illustrate the useful insights offered by a simple model. It is well known that the preference for neighborhood of an individual may depend on variety of factors such as, income, language, religion, race, age etc. Instead of considering all these factors in model, Schelling [29] posits that societal segregation may result from an individual being aware of her neighborhood and exhibiting slight preference for her group. To demonstrate this, he developed an agent based model of societal segregation. In this simplified model the behavior of an agent is governed by the proportion of agents like her in her neighborhood, and the same threshold for tolerance is used for each agent. However, in real life everyone might have different threshold and many other factors may govern an individual’s neighborhood selection. Despite its simplicity, using this model Schelling [29] was able to generate the pattern of segregation that is empirically observed in urban areas [30]. We take similar approach to develop a typical model of reader behavior in our agent based model. Our reader representation is simple, but we are able to capture the salient features of article popularity evolution.

The arrival of readers is modeled sequentially, and they start their reading activity from the front page. The number of links a reader follows before she stops reading is modeled in the standard way as a random variable *L* drawn from an inverse Gaussian distribution [31]. Mathematically, the distribution *L* is given by,

$$P(L)=e^{-\frac{\lambda {\left(L-\mu \right)}^{2}}{2L\mu^{2}}}\sqrt{\frac{\lambda }{2\pi L^{3}}}$$ (2)

In Eq 2, *μ* and *λ* are the mean and shape parameter respectively. The average page views per visitor for news websites was obtained from SimilarWeb (similarweb.com), and for the variance we use values consistent with previous empirical findings on Web and mobile surfing [31, 32]. Readers use different selection mechanisms of articles for the front page and the category specific pages. See S1 Algorithm in S1 Appendix for the implementation of the reader model.

The system representing the *Media Manager* maintains a database of the comprehensive list of articles. Let *CL*_*i*_ denote the comprehensive list of articles corresponding to *i*^*th*^ news category. Then (*CL* = U_*i*_*CL*_*i*_), denotes the set of all articles. From *CL*, the algorithm selects *N* articles for display as “recommendations” at each time index ’*t*’. Generally, recommendations are offered in personalized or non-personalized way [33]. We focus on non-personalized top-N news recommendations. Articles are sorted and those with the highest counts are selected for the Display List (*DL*), which is the “top-N” recommendations.

Unlike the typical top-N recommender, in FNRS articles are selected according to probability:

$$p_{a}(t)=\frac{c_{a}^{\gamma }(t)}{\sum_{j}c_{j}^{\gamma }(t)}$$ (3)

where c_*a*_(*t*), represents the click counts of an article ‘*a*’ at a given time *t*, and $\sum_{j}c_{j}^{\gamma }(t)$ therefore represents, at time *t*, the sum of amplified counts of articles (those are not yet selected for DL). These probabilities are used for sampling without replacement (since articles selected into DL are no longer considered), and repeated N times to generate N recommendations in DL. Importantly, media managers can choose the desired exponent (*γ*) to guide self-organizing outcomes. This seemingly simple choice of a single parameter can drive a whole range of self-organizing behavior, each leading to very different emergent outcomes.

The media manager also oversees the *Adaptation* of the news Web site, in response to arrival of new articles. Upon arrival of a new article, *CL* is updated with the necessary details. Three hyperlinks for the newly arrived article are created, and they are inserted in three different lists in the system. On the front page, the hyperlinks appear at the top position of Breaking News and article-specific category sections and at the other Web pages (except front) it appears at the top position of the chronological listing accessible through category links provided on the top of that particular web page. The copies of the oldest article from each of these lists is removed and the article’s status is updated in *CL*_*i*_.

### Interaction

We use a "read-index" in the reader model (S1 Algorithm in S1 Appendix) to assign a higher weight of reading probability for the most recent articles in all lists that follow the chronological order of display (i.e. except the most-popular list). Although there can be various ways to assign higher reading probability to the top position in the display list [34], we implement it using the probability function $r_{i}=\frac{N+1-i}{\sum_{i=1}^{N}i}$ for a particular article with rank *i*,*i* ∈ {1,2,…*N*} in a given list.

The interaction of a focal agent (reader) with other agents primarily takes place indirectly through the top-N (or trending) list (because this list displays the articles which are also being prominently read by other readers in the system). Other possible form of interaction among readers is also possible through parallel activities and sharing of articles through social media platform. For the ease of exposition, these complexities are excluded from the current model.

### Algorithm 1. Selection of articles for recommendation using feedback function

1. pick ← 0; P ← K

2. i. $\Omega =\left\{\begin{array}{ccc} c_{1}, & c_{2}, & c_{3},\cdots \;c_{P} \end{array}\right\}={\left\{c_{i}\right\}}_{i=1}^{P}$

 ii. $\Omega_{\gamma }=\left\{\begin{array}{ccc} c_{1}^{\gamma }, & c_{2}^{\gamma }, & c_{3}^{\gamma },\cdots \;c_{P}^{\gamma } \end{array}\right\}={\left\{c_{i}^{\gamma }\right\}}_{i=1}^{P}$

 iii. $\Omega_{\gamma }^{’}=\left\{\begin{array}{ccc} c_{1}^{\gamma }, & {c_{1}^{\gamma }+c}_{2}^{\gamma }, & {c_{1}^{\gamma }+c}_{2}^{\gamma }+c_{3}^{\gamma },\cdots \;\sum_{k=1}^{P}c_{k}^{\gamma } \end{array}\right\}={\left\{\sum_{k=1}^{i}c_{k}^{\gamma }\right\}}_{i=1}^{P};1\leq k\leq i$

 iv. $\Omega_{\gamma }^{0}=\left\{\begin{array}{cc} 0, & {\left\{\sum_{k=1}^{i}c_{k}^{\gamma }\right\}}_{i=1}^{P} \end{array}\right\}={\left\{S_{\gamma }^{0}\left(i\right)\right\}}_{i=0}^{P}$

 v. Generate a random integer R, such that $R\in \left[\begin{array}{cc} 1, & S_{\gamma }^{0}\left(P\right) \end{array}\right]$

 vi. Find index *i*, such that $R\in \left(\left.\begin{array}{cc} S_{\gamma }^{0}\left(i-1\right), & S_{\gamma }^{0}\left(i\right) \end{array}\right]\right.$

 vii. Select article *i*, for recommendation in FNRS

3. pick ← pick + 1

4. Remove article *i* from sorted set Ω

5. Continue from Step 2(i) until: pick <*N*

### Illustration of update process

The key task in the design of FNRS is the selection of N articles as recommendation using the feedback probability function (i.e., the specific implementation of Eq 3 where article selection probabilities are based on the proportions). Suppose there are K recent articles in the system that have a chance to appear in the recommended list, and Ω denotes the sorted set of these articles. At a given time index *j*, *c*_*i*_ denotes the click counts that an article *i* has received. Using these notations Algorithm 1 presents the steps involved in selection of N articles in FNRS.

## Results

We present results from running the agent-based model based on the comparison between FNRS and the top-N NRS using the measures defined below. The results are mainly illustrative, to show how the feedback parameter can help guide different emergent outcomes.

For the results corresponding to different feedback parameters *γ*, the counts of articles in *DL* were updated *in parallel* in both mechanisms: FNRS and the top-N system. Specifically, as the agent based simulation runs, from the same initial conditions we run two parallel universes (two parallel simulations) under different selection mechanisms to keep track of the exact count differences. This ensures that, other than the selection mechanisms, all else remains the same in these parallel universes that enables comparison of the emergent outcomes.

### Measures

The first measure focuses on how *minor* differences get amplified at the boundary due to feedback and is based on examining the count evolution of the N^th^ article versus the (N+1)^st^ article in a top-N list. The second measure focuses more generally on how the top-N list is reinforced due to feedback. Finally, the third measure is Gini which captures how feedback increases clicks for all popular articles at the potential expense of increasing click-count inequality among articles. The definitions are below.

#### Measure 1 (recommendation boundary amplification)

This measure is introduced in [2] to study the impact of NRS on boundary amplification in a very simple setup. It is defined using the ratio of clicks of *N*^*th*^ and (*N* + 1)^*th*^ article, in DL and we denote it as M1. These two articles were marked in the beginning of the simulation and were tracked over the simulation. M1 is defined as the logarithmic-ratio of the clicks of *N*^*th*^ and (*N* + 1)^*th*^ articles at each time-index (*j*) of the simulation. That is at the *j*^*th*^ iteration of the simulation, for a given FNRS, $M1(\gamma ;\;j)=\ln\left({click}_{Nj}\right)-\ln\left({click}_{\left(N+1\right)j}\right)=\ln\left(\frac{{click}_{Nj}}{{click}_{\left(N+1\right)j}}\right)$. This measures the relative change in clicks of *N*^*th*^ and (*N* + 1)^*th*^ article. At the start of the simulation, *click*(*N*) ~ *click*(*N* + 1), hence *M*1(0)~0. Particularly in the case of hard-cutoff recommendation, this measure helps us demonstrate the presence of Butterfly effect [35] and the lack of individual fairness [36] among N^th^ and (N+1)^th^ articles.

#### Measure 2 (top-N reinforcement)

We introduce this metric and it is measured in the following way. For any given recommender we measure the percentage of new clicks (i.e., after *j* = 0), received by the articles outside the *initial* top-10 list—determined through sorted click-counts. This measure is denoted by M2 and is defined as,

$$M2\left(\gamma ;\;j\right)=\left\{1-\left(\frac{\sum_{top-10\;list}\left(\#new\;clicks_{j}\right)}{\#\;total\;new\;clicks_{j}}\right)\right\}*100$$

This is a measure that is interesting particularly when viewed over time. In systems where there is reinforcement in the top-N list, we would expect to see this measure decrease over time towards its value in a hard-cutoff scenario (because more of the new clicks into DL go to the top-N articles). The slope of this can also provide information on the speed at which this effect materializes. This measure therefore helps us examine the reinforcement behavior of FNRS for the different choice of the feedback value *γ*. As an extreme case when there is a high likelihood of only reading articles in the recommended list (*probability of reading an article from DL~1*), then under the top-N (hard cut-off) selection we would expect M2 to be zero since all new clicks will go to the same articles in the top-N list.

##### Gini

It is one of the popular measures for quantifying inequality in a system [37]. To assess the impact of news recommendation on overall popularity inequality we also track this metric during the simulation. Gini index is defined as absolute mean difference divided by mean of the click counts of articles. Mathematically, Gini coefficient is defined in the following way,

$$G=\frac{\sum_{i=1}^{P}\sum_{j=1}^{P}|C_{i}-C_{j}|}{2P^{2}\bar{C}}$$ (4)

In the above expression (4), *P* represents the number of articles in the system at any given time, and $\overset{-}{C}=\frac{1}{P}\sum_{i=1}^{P}C_{i}$. Note that the minimum value of G is 0 and it happens in an egalitarian system where all articles receive same number of clicks. An in the most unequal system, one article receives all clicks and other articles receive none. In this extreme case $G=\left(1-\frac{1}{P}\right)$, that is *G* ~1 displays high inequality.

##### The update rule

At each time step, one reader arrives, and the arrival of a new article is determined by the rate parameter of exponential distribution. Upon arrival of a reader, *apriori*, the number of articles to be read by the reader is determined. The reader proceeds reading articles from front page, per her behavior described in S1 Algorithm in S1 Appendix. The probabilities for the selection of different articles from a reader are controlled in the simulation. If the reader selects an article, then the count of the selected article is increased by 1. The order of display of articles (both chronologically and based on popularity) are updated at each time index.

For two different NRS, top-N and FNRS, the selection of *N* articles is made for the recommended display list (DL), and DL is updated at each time index. To the extent possible, we selected realistic values of simulation parameters, and for this purpose we gathered information from various sources. We provide a detailed discussion on the choice of different simulation parameters in S1 Appendix.

#### Simulation parameters

Usually the popularity of items online follows the power-law (Zipf) distribution [38], therefore before the simulation starts we generated 400 articles (50 articles corresponding to each category), and choose the interval of (1, 1000) to assign them initial popularity. We take average of the number of desktop visitors on different news websites in the sample to determine *μ* (see S1 Table in S1 Appendix for details).

We study the behavior of FNRS for different choices of the feedback exponent *γ*. In the simulation, it has been varied with different integer values between 1,2,….10. For each of the cases, in the beginning of the simulation we use the same seed values for the generation of random numbers as well for more accurate comparisons across runs.

To account for the prominence of most-popular articles, the higher values of the reading probabilities are of interest. Also, empirically it has been observed that recommendation engines usually exhibit strong influence on users [39, 40]. Though different combinations of *p*,*q*,*r* were used in the simulation as given in Table 2, we develop our discussion on a particular choice of (*p*,*q*,*r*) = (0.7,0.2,0.1). This particular combination helps us examine the case where, FNRS has mild influence on reading behavior, for the other cases, in which, FNRS displays even stronger influence on reading behavior will easily follow from the discussion in present research. Additional simulation results for few selected cases are reported in S1 Appendix. To the extent readers exhibit moderate to high preference for the recommended and recent articles, the findings discussed below do not change.

**Table 2:** Simulation parameters.

Zipf exponent (*s*)	1.4
Initial seed interval for 400 articles	(1,1000)
Number of articles in DL	10
*μ*	3.2
*λ*	3
Number of readers	50,000
Arrival rate of News articles (*ρ*)	0.003
(*p*,*q*,*r*)	(0.3, 0.4, 0.3), (0.3, 0.5, 0.2), (0.4, 0.3, 0.3), (0.4, 0.4, 0.2)
	(0.5, 0.3, 0.2), (0.6, 0.2, 0.2), (0.7, 0.2, 0.1), (0.8, 0.1, 0.1)

We choose number of readers to be 50,000 in the simulation to ensure that this number is a reasonable estimate to: (a) demonstrate the emergent aspects of news recommendation, (b) on average dynamic update of the feedback exponent is performed at an interval of 20–30 minutes in a real system, and (c) the reported number is well within the daily visitors to a typical news website.

#### Properties of FNRS

**Fig 2:**
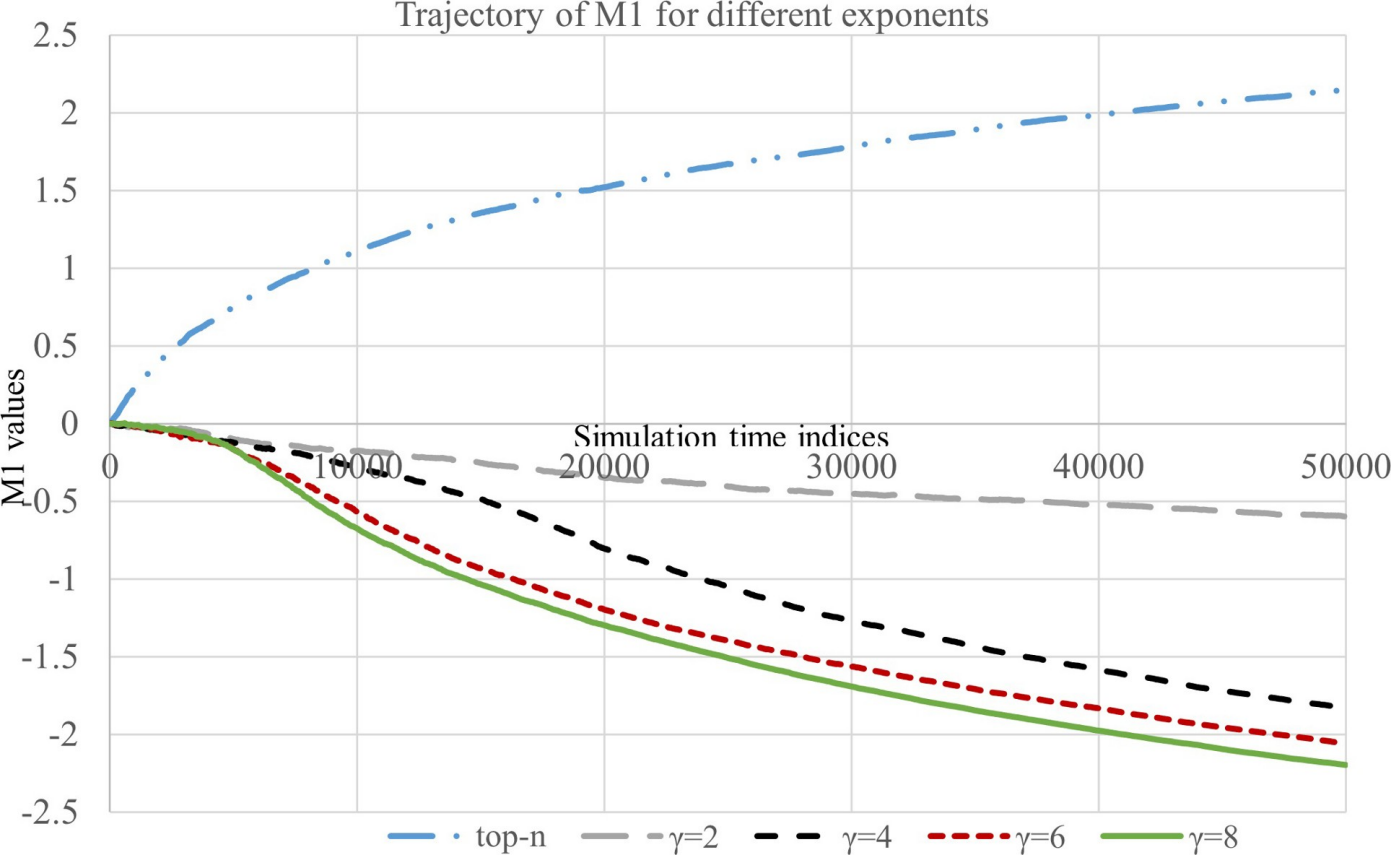
Boundary amplification of articles.

**Fig 3:**
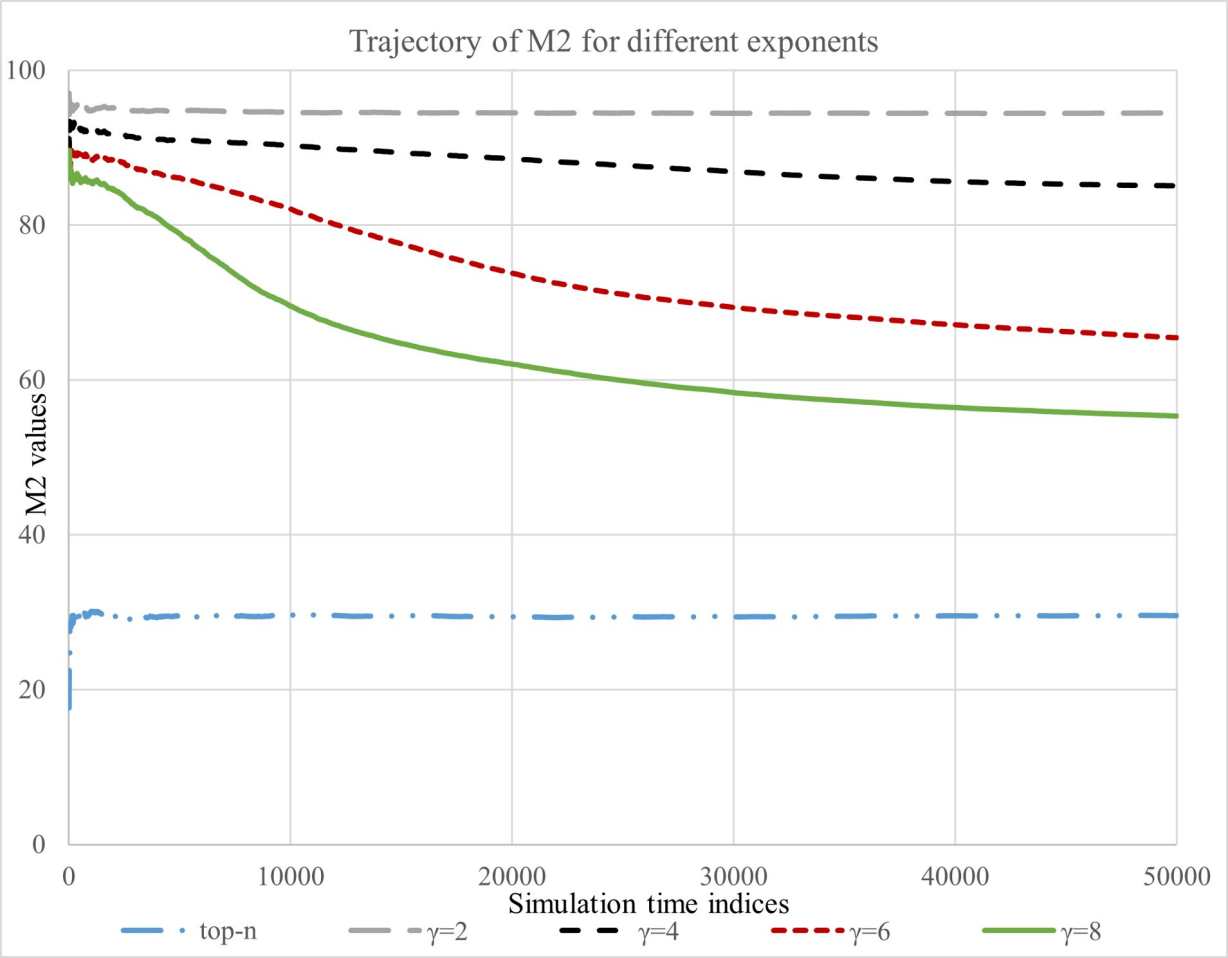
Reinforcement behavior of FNRS.

**Fig 4:**
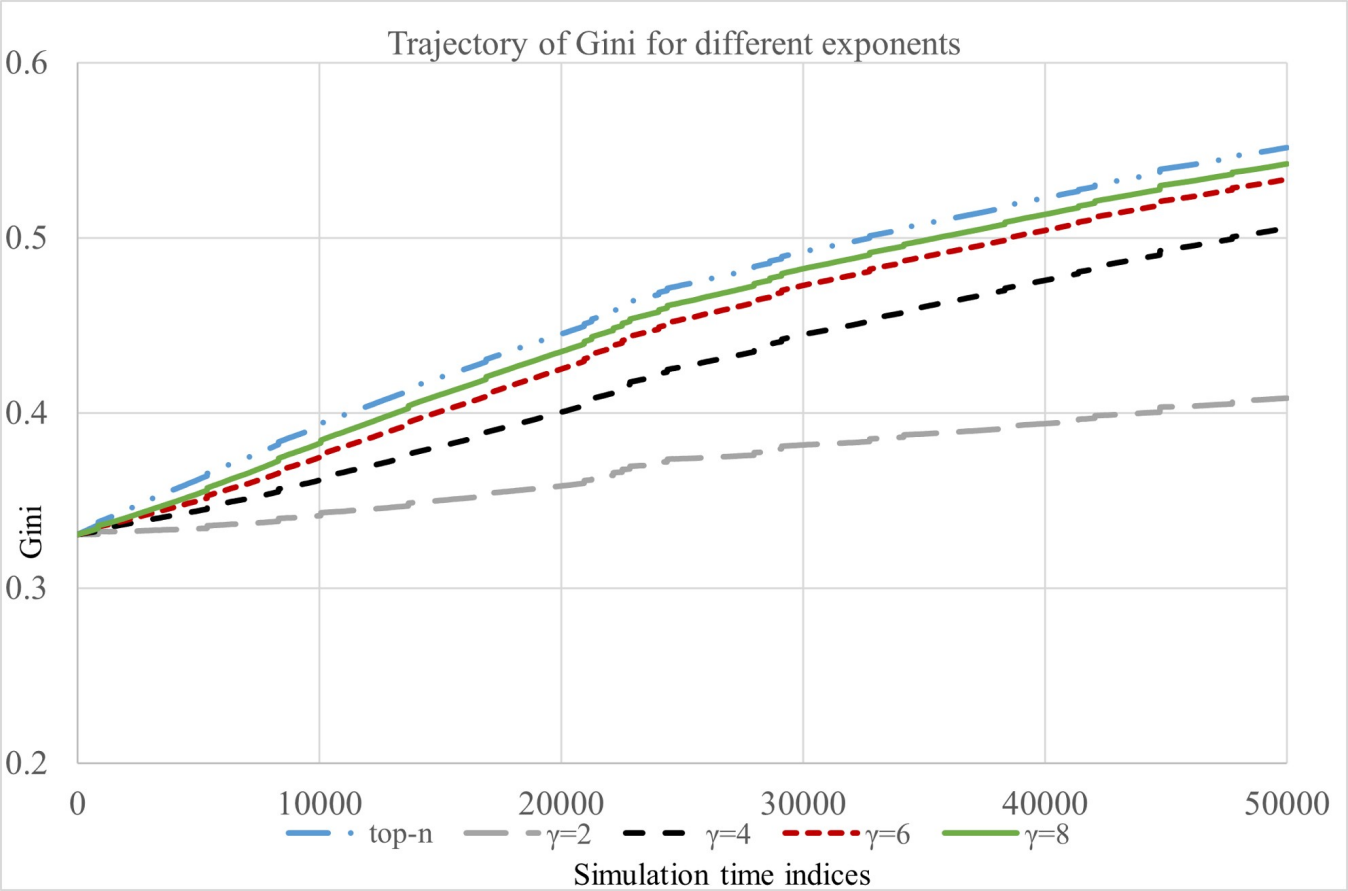
Inequality in popularity measured using Gini index.

The trajectories of M1, M2 and Gini is presented in Figs 2–4, respectively. First, we summarize our findings based on the measure M1.

It can be observed from the path followed by M1 (Fig 2) that for an influential top-N NRS the initial difference between the counts of *N*^*th*^ and (*N* + 1)^*th*^ article gets amplified heavily and quickly. As expected, for *γ* = 2, 4, 6, 8 the measure M1 takes values significantly lesser than the top-N recommendation that uses hard cutoff (see Fig 2 and additional results in S1 Appendix). It is also noteworthy that in the case of FNRSs, the popularity amplification trend between Nth and (N+1)th article does not follow a deterministic trend because their initial click count is almost same. Therefore, Nth and (N+1)th articles compete for the recommendation spot over the initial period of simulation. In the presence of positive feedback one among them eventually attains a significant lead over the other and gets selected for recommendation deterministically. The extent of popularity amplification between the Nth and (N+1)th article is controlled by the feedback exponent. These findings suggest that the phenomenon of artificial amplification due to top-N selection can be significantly mitigated using FNRS, and hence recommendation using feedback is more suitable to maintain individual fairness [36].

Fig 3 presents the findings based on M2. In the figure, the bottom line corresponds to the top-N NRS (hard-cutoff). We observe that as the value of *γ* increases, the performance of FNRS becomes closer to the top-N NRS. Further, the FNRS with positive feedback eventually may behave as top-N after sufficient number of time steps (for example see the path of *γ* = 8). Hence, using feedback selection mechanism we can generate behavior similar to traditional top-N recommendation, while maintaining reasonable level of diversity in the selection of articles.

We also observed that the share of new click counts received by the articles which were not in the initial (*t* = 0) recommendation list also depends on the preference a reader exhibits for the recommended articles. For example, after sufficient time steps in S7 Fig in S1 Appendix approx. 70% of new Web traffic is received by the non-recommended articles, whereas in S9 Fig in S1 Appendix it is approx. 50%. In the case of FNRS for a given feedback exponent, as recommendation evolves over time a higher level of reinforcement is observed if readers exhibit higher preference for the recommended articles.

Finally, Gini measures the extent of inequality in popularity of articles induced by the recommendation process. We observe (Fig 4) that the recommendation that uses sorting of articles based their click counts (hard cutoff) induces highest level of inequality in the popularity of articles as the simulation progresses. When readers exhibit moderate to high preference for the recommended articles the Gini coefficient exhibits increasing trend with respect to: (a) simulation time steps, (b) extent of positive feedback, and (c) the preference for the recommended articles by readers (see Fig 4, and S1–S3 Figs in S1 Appendix).

For the robustness check of the findings related to Gini coefficient we replicated the whole simulation process 50 times with different initial seed values and recorded the Gini coefficient corresponding to each feedback exponent (*γ*) at the end of each simulation replication.

We performed two sample t-test for independent samples for the following cases (*γ*_*a*_, *γ*_*b*_) = (2, 4), (4, 6), (6, 8) *and* (8, ∞) with *γ*_*a*_ < *γ*_*b*_. Here *γ*_*b*_ = ∞ corresponds to the implementation of top-n recommendation. As stated earlier, we expect that a higher value of feedback exponent will cause more inequality in popularity of articles, therefore a higher Gini coefficient will be observed. Following hypothesis was tested corresponding to each of the cases mentioned above.

$$H_{0}:G_{\gamma_{a}}=G_{\gamma_{b}}\;vs\;H_{a}:G_{\gamma_{a}}<G_{\gamma_{b}};\;\left(\gamma_{a}<\gamma_{b}\right)$$

In other words, we are interested in examining if a higher value of feedback exponent results in a higher level of inequality in click counts of articles. To conduct the two sample t-test, we first examined the equality of variances for all cases of two independent samples using F-test. We failed to reject the null hypothesis of equal population variances (at 0.05 significance) in all cases (see Table 3). Therefore, we conducted the two-sample t-test for independent samples with equal variances. In all cases we reject the null hypothesis (*H*_*0*_). Therefore, we conclude that a higher value of exponent leads to a higher level of inequality in the click counts of articles.

**Table 3:** Summary of hypotheses tests two-independent samples (sample size = 50).

(*γ*_*a*_,*γ*_*b*_)	(2, 4)	(4, 6)	(6, 8)	(8, ∞)
F-test for equality of variance (p-value)	0.134	0.051	0.409	0.446
t-static (p-value) for independent sample test with equal variances	94.11 (0.00)	34.93 (0.00)	12.65 (0.00)	13.13 (0.00)

## Dynamic adaptation of FNRS

### Extending M1 & M2 as design principles of FNRS

Thus far we have shown how a simple feedback parameter in FNRS can be elegantly used to mitigate some potentially harmful impacts of NRS. While this is an important contribution of our work, it does not provide a media manager sufficient flexibility to *continuously* guide the adaptation of system in a changing news readership landscape. This is the issue we turn to here.

As a case in point, if we retain a feedback exponent (*γ; such that γ > 1*) over an extended period of time, the NRS will drift towards an “equilibrium” state, where only few articles will receive majority of clicks (akin to top-N). From the complexity perspective, these states are considered “dead” or uninteresting [12]. From a practical perspective, media managers would rarely want their extensive news ecosystem to focus on a handful of news articles that most users read to the detriment of much of the other important news curated by their team of journalists and editors. To enable FNRS to adapt to the changing environment, in this section we develop a framework for updating the feedback exponent at selected discrete points in time, albeit with some form of augmentation or human intervention. In the complex systems perspective, the exponent is used as the prescription (or strategy) for the collective behavior of the system, while the sampling points act as lever points (or corrective policy intervention) at the critical stages of emergence that allow the system to co-evolve with environment and to respond based on extant capabilities [12]. The environment is not a constant, but changing and often co-evolving with time [41] as well.

An adaptive FNRS would balance two major challenges encountered in recommendations [12]: exploration (acquisition of new information and capabilities), and exploitation (the efficient use of information and capabilities already available). To perform the task of exploitation, we need to determine the extent to which we want to: (a) generate less distortion in recommendation by giving some chance to *less* popular articles in the recommended list, (b) capitalize on popularity of articles corresponding to highest click by recommending them (top-N reinforcement), and (c) the stopping point until we want FNRS to exhibit same behavior. We illustrate the path followed by the first two phenomena in simulation (their trajectory) through metrics M1 & M2. We examined the performance of FNRS for different values of *γ*, over a fixed number of readers (or horizon; *t* = 50,000) for these metrics. Further, for M1 we only focus on boundary articles: N^th^ and (N+1)^st^. To extend these concepts for all articles in the system, we need to modify the functional form of M1 & M2.

Articles getting a position in the top-N list receive substantially higher visibility than the other articles. M1 captures this notion well for the boundary articles. By the inherent design of top-N recommendations, the extent of clicks for other recommended articles will at least be same if not higher than the N^th^ article (see the empirical evidences reported in [42, 43]). Promoting the most-read articles directs more attention to them, and hence create click-share *distortion* in the underlying distribution of article clicks over time. This can have its longer-term consequences for the news site, where only few selected articles receive majority of the new clicks. In an environment, where news industries are struggling to resurface or repackage their huge archives of news to readers [44], introduce diversity in recommendation [10], and trying to engineer serendipity in their recommendation engines [45], the display of articles based on hard-cutoff can be potentially troublesome due to susceptibility of the top-N recommender in creating distortion in the popularity of articles. Here distortion is defined as increase in the click-count share of the popular articles and decrease in the click-count share of the less popular articles over time due to recommendation. Hence, one objective for the dynamic adaption of FNRS is to have low distortion (defined formally later in this section), which generalizes the M1 measure.

M2 measures the extent of top-N reinforcement. The higher the feedback exponent, the higher the chance that popular articles will appear as recommendation. The more the trajectory of M2 is closer to the trajectory of hard-cutoff recommendation (Fig 3), the more FNRS exhibits recommendation akin to hard-cutoff. If clicks (or views) of articles are considered as a quality surrogate for recommendation, then the hard-cutoff top-N recommender has high *accuracy* since it only picks articles with highest counts. Managers wishing to optimize near-term revenue goals may find this attractive since prominently highlighting popular articles in a top-N list can drive even more traffic to these articles from newer viewers. On the other hand, the probabilistic recommender that recommends articles proportional to their popularity (*γ* = 1), is true towards the "natural share" of articles but often recommends articles that are not the ones with highest counts, and hence sacrifices “accuracy”. Hence, a second objective for the dynamic adaption of FNRS is to have high accuracy, which generalizes the M2 measure.

### Conflicting objectives in design of FNRS

We formalize these two trade-offs (accuracy and distortion) in a formal setup. Since the FNRS can operate in a broader continuum compared to the other recommendation methods, it is particularly effective as a method to make a choice between these two trade-offs. The choice between the level of accuracy and distortion in a FNRS has the notion of subjectivity based on a media manager’s preference for these two metrics.

We record the clicks received from the initial set of readers that arrive in the system over a pre-determined time interval say (*t*_0_,*t*_1_). Observation during this initial period, helps us to stabilize the initial randomness in click evolution process of articles. At *t*_1_, we select another time interval (*t*_1_, *t*_2_) with discrete indices *j’s* (*t*_0_ < *t*_1_ ≤ *j* ≤ *t*_2_) to discuss the following novel constructs.

#### Accuracy-loss (generalization of M2)

At any given time for FNRS we define accuracy-loss based on comparison to a benchmark of distribution of counts of articles that appear as recommendations in a top-N NRS at the exact same time index. To quantify accuracy-loss due to recommendations, we assume that the count distribution of articles, when they are selected corresponding to highest count in top-N recommendation at each time index represents the "ground truth". We measure accuracy-loss for an FNRS with a given exponent *γ*, by implementing two parallel universes (systems) from the same initial conditions—FNRS with the specific level of a feedback exponent, and the other based on a traditional top-N list. In this manner, we define accuracy-loss metric in the following way at time index *j*:

$$accuracyloss\left(E_{j}^{\gamma }\right)=\text{ln}\frac{\sum_{i=1}^{N}C_{ij}^{H}}{\sum_{i=1}^{N}C_{ij}^{\gamma }}$$ (5)

In Eq 5, *N* represents, the number of articles appearing in top-N (or probabilistic) “recommended” list. $C_{ij}^{H}$ represents the clicks of *i*^*th*^ article, appearing in the top-N (hard-cutoff) NRS, at the *j*^*th*^ time index. Whereas, $C_{ij}^{\gamma }$ represents the clicks of *i*^*th*^ article appearing in the FNRS with exponent *γ*, at the *j*^*th*^ time index. Hence, $\sum_{i=1}^{N}C_{ij}^{H}$ and $\sum_{i=1}^{N}C_{ij}^{\gamma }$ represent the sum of clicks of all articles that appear in traditional top-N NRS and FNRS with exponent *γ*, respectively, at the *j*^*th*^ time index as recommendation.

As the simulation progresses, we average the accuracy loss over the number of simulation time steps at which the recommended lists are updated to simplify the expression without introducing significant error. Hence, the average accuracy loss is:

$${\bar{E}}_{l}^{\gamma }=\frac{1}{\Delta t}\sum_{j=t_{1}}^{l}\overset{\gamma }{\underset{j}{E}}=\frac{1}{\Delta t}\sum_{j=t_{1}}^{l}\text{ln}\frac{\sum_{i=1}^{N}C_{ij}^{H}}{\sum_{i=1}^{N}C_{ij}^{\gamma }};t_{1}\leq j\leq l\leq t_{2},\Delta t=l-t_{1}$$ (6)

Where *ℓ* is a time-index between (*t*_1_, *t*_2_), and we use it as a subscript for the average value of a metric over the interval (*t*_1_, *ℓ*).

#### Distortion

M1 illustrates the extent of amplification due to recommendation, albeit only for the boundary articles. We extend this notion for overall count amplification due to recommendation, measured by the metric *distortion*. To measure share distortion, we assume that the initial share of articles at *t*_1_, before recommendation starts, is the "ground truth".

To quantify distortion, we measure the distance between the probability distribution of initial share of articles in the system at *t*_1_, and the modified share of articles after the recommendation, using Jensen-Shannon Divergence (JSD) [46]. Distortion created in the natural share of articles due to FNRS with exponent *γ*, is averaged over the number of simulation time steps at which accuracy-loss metric are updated (Eq 6). Note that the definition of JSD metric to measure the distance between two distributions remains valid even in the presence of retiring of old articles and arrival of new articles.

Let us denote the probability distribution of articles in the system in the presence of FNRS with exponent *γ*, at the time index *j* as $q_{j}^{\gamma }$ and the initial share (*j* = *t*_1_) of articles as probability distribution *p*. Then mean JSD is,

$$distortion\left({\bar{JS}}_{l}^{\gamma }\right)=\frac{1}{\Delta t}\sum_{j=t_{1}}^{l}\text{JSD}\left(p,q_{j}^{\gamma }\right);t_{1}\leq j\leq l\leq t_{2},\Delta t=l-t_{1}$$ (7)

Both objectives (Eqs 6 and 7) depend on highly complex and random mechanism of readership, arrival of new articles, recommendation, and update of the system. Also, the trade-off between minimizing accuracy-loss and distortion would require a manager’s subjective decision. Hence, the classical optimization techniques of “tame” problems will fail to offer policy choices [19].

These kind of planning problems which are result of the complex interaction of various components, constantly changing environment, and has presence of multiple stakeholders and perspective, can be categorized as a “wicked” problem [20, 21]. The objectives of wicked problems are incomplete, contradictory, changing over time, and they are evaluated based on subjective preferences. Due to the conflicting nature of multiple objectives, wicked problems can be assisted through a set of Pareto-optimal solutions [47].

To generate Pareto-optimal solutions we present a framework for the evolutionary multi-objective optimization that offers sufficient flexibility and reasonably encompasses the complex search spaces and ill-behaved objective functions [48]. Evolutionary algorithms inspired by natural selection have been used to generate high-quality solutions to difficult problems with highly complex search spaces and multiple conflicting objectives [48]. In our context, we use the evolutionary algorithm NSGAII [49], to explore the search space of accuracy-distortion trade-off.

### Multiobjective Pareto-optimal solutions

Consider a multi-objective optimization problem with two conflicting objectives (*f*_1_(*z*),*f*_2_(*z*)), *z* being a vector of decision variables. For each of the two conflicting objectives (*f*_1_(*z*),*f*_2_(*z*)), there exists one optimal objective value and the corresponding different optimal solution(s). Because, the minimum solution of objective functions *f*_1_(*z*) and *f*_2_(*z*), need not be the same solution, in general, we encounter non-existent solutions in multi-objective optimization [49]. To overcome this issue, in the presence of multiple conflicting objectives we use Pareto-optimal (non-dominated) solutions. In our context, the solution *z* is a vector of two decision variables, namely, *γ* and *ℓ* and the objective vector *F*(*z*) is defined as,

$$F\left(z\right)=\left[f_{1}\left(z\right)={\overset{-}{E}}_{l}^{\gamma },f_{2}\left(z\right)={\overset{-}{JS}}_{l}^{\gamma }\right]$$ (8)

We minimize the objective vector (8), subject to:

$$4\leq \gamma \leq 10;t_{1}\leq l\leq t_{2}$$ (9)

Our goal is to find a set of solution vectors, z* = [γ*, j*] that are Pareto-optimal with regard to objective vector (8) and satisfy constraint vector (9). It is also noteworthy that the objective vector *F*(*z*), also depends on probability distribution of reading activities. But this is an exogenous factor and depends on reading behavior of visitors, hence it does not appear in the decision variable of optimization function *F*(*z*).

**Fig 5:**
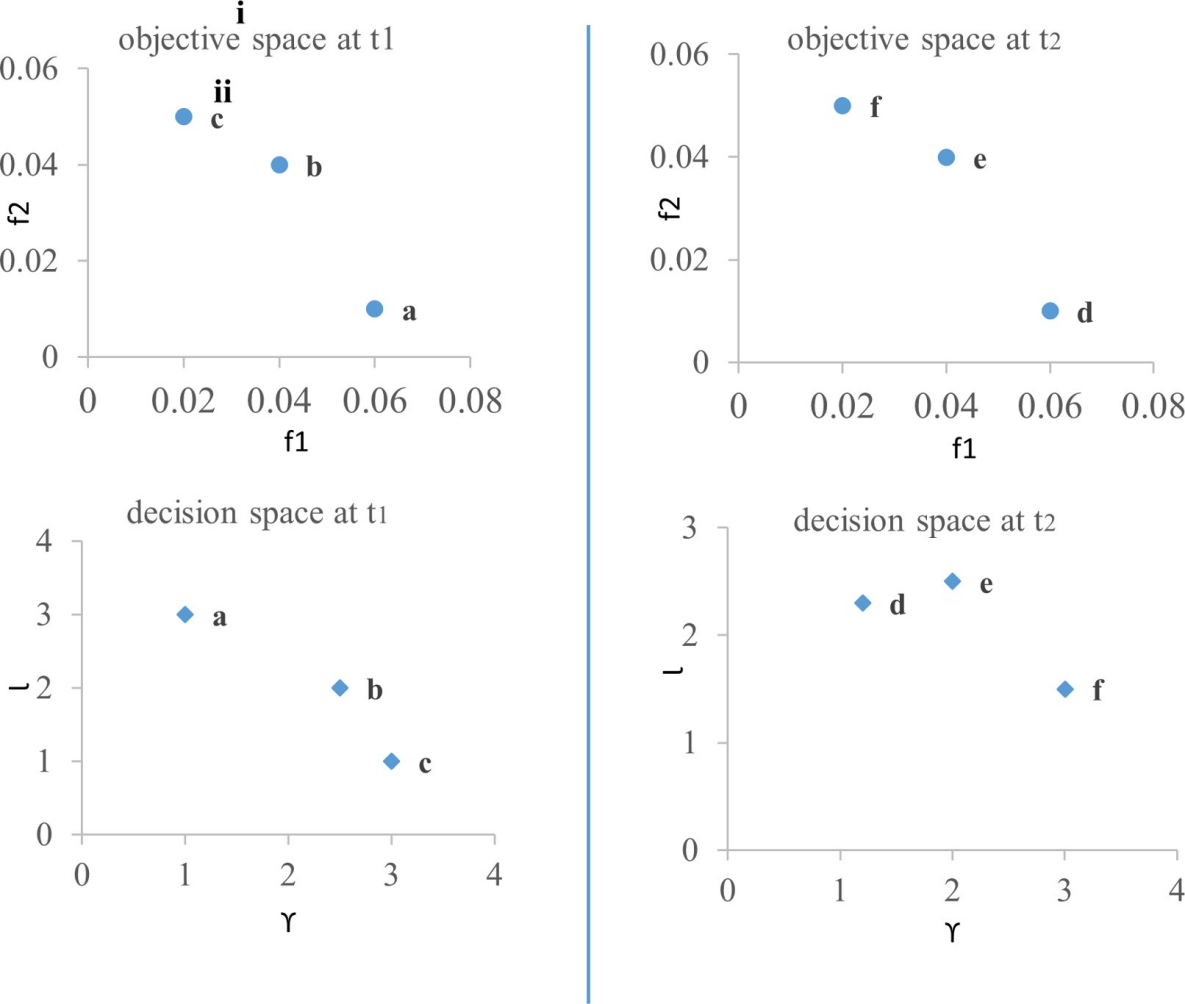
Illustration of non-dominated solutions. (i) Set of non-dominated solutions (upper panel) and the value of their corresponding decision variables (lower panel) at t1. (ii) Set of non-dominated solutions (upper panel) and the value of their corresponding decision variables (lower panel) at t2.

For illustration, consider the non-dominated solutions depicted in Fig 5(i). For a given solution say ‘a’ in the objective space, its corresponding solution is shown in decision space with same character (i.e. ‘a’) at a given time *t*_1_. In this example, if manager chooses the non-dominated solution ‘a’ at *t*_1_, then the corresponding feedback parameter is *γ* = 1, and the stopping point is 3 units of time (say, 3*x*) beyond *t*_1_. Hence, she uses *γ* = 1 in the interval [*t*_1_,*t*_1_ + 3*x*].

### Dynamic adaptation of FNRS

As mentioned earlier the use of a feedback exponent (*γ* > 1), over the same set of articles with high clicks over an extended period will render FNRS to emulate top-N recommendation and hence does not offer much flexibility to adapt the system with changing environment. On a typical news website, there will be arrival and retiring of articles, random and often turbulent reading patterns–partly driven by social media traffic. An FNRS should be flexible enough to adapt and co-evolve with the changing environment [12].

To model FNRS as an intelligently adapting system, we consider two decision variables—(a) the feedback exponent *γ*_*t*_, and (b) the time-interval (or stopping point; Δ*t*) corresponding to the current exponent value. The adaptation of FNRS can be represented as a search for relative optima over a “rugged landscape” of these two decision variables. For any combination of (*γ*_*t*_, Δ*t*), *γ*_*t*_ will be used for the time interval (*t*,*t* + Δ*t*). At *t’* = *t* + Δ*t*, a new combination of (*γ*_*t’*_,Δ*t’*) are chosen from the Pareto-optimal solutions (exploration) to update the feedback exponent and the stopping point. For illustration see Fig 5(ii), in which a manager may prefer to choose non-dominated solution ‘e’. Corresponding to solution ‘e’, the feedback parameter is *γ* = 2, and the stopping point is 2.5 units of time beyond *t*_1_ +3*x*. Hence, she uses *γ* = 2 in the interval [*t*_1_ + 3*x*, *t*_1_ + 5.5*x*].

### Algorithm 2: Pseudo code to dynamically update the exponent *γ*

a. At *t*_0_ = 0, choose time interval [*t*_0_, *t*_1_] over which the clicks received by the articles will be observed without recommendation

b. Let *t* = *t*_2_(*t*_2_>*t*_1_)

c. Perform following steps at each *t*, to update the exponent values *γ*

 i. Update the simulation parameters using most recent observations corresponding to the moving time window [*t*– *δt*,*t*]

 ii. Determine the Pareto optimal front for accuracy-loss and distortion values using NSGAII

 iii. Choose the desired value of the control exponent (*γ*_*t*_) and the corresponding time interval say, $\hat{\Delta }t$ from the Pareto front

 iv. $t=t+\hat{\Delta }t$

 v Use *γt* in FNRS until *t*

The update process for (*γ*_*t*_, Δ*t*), gives system the characteristic of “evolving structure” with changing environment, and serves as a credit assignment procedure [12] at intermittent time points over a desired interval. Selection of articles using updated system parameters and the feedback exponent reward those articles that seem to be causing better performance, and can be seen as corrective action [12]–subject to manager’s preference. The update process enables FNRS to make a balance between exploration (update of (*γ*_*t*_, Δ*t*) based on newly acquired information, and exploitation (the efficient use of Pareto-optimal solutions for recommendation). For the generation of Pareto-optimal solutions we do not have a definitive stopping rule, and due to constant update process the objective functions have the property of “moving target” [12].

To determine the Pareto optimal front for accuracy-loss and distortion we use NSGAII. NSGAII is an Elitist Non-Dominated Sorting Genetic Algorithm that generates various Pareto-optimal solutions in a single run of the algorithm. The selection operator of NSGAII selects a population of solutions in such a way that it preserves both diversity and elitism of non-dominated parent and offspring solutions. The general update process of exponents is detailed in Algorithm 2. In the pseudo-code, steps c(i-iv) can be considered as exploration and the step c(v) as the exploitation step of the adaptation process.

### Simulation results using NSGAII

**Fig 6:**
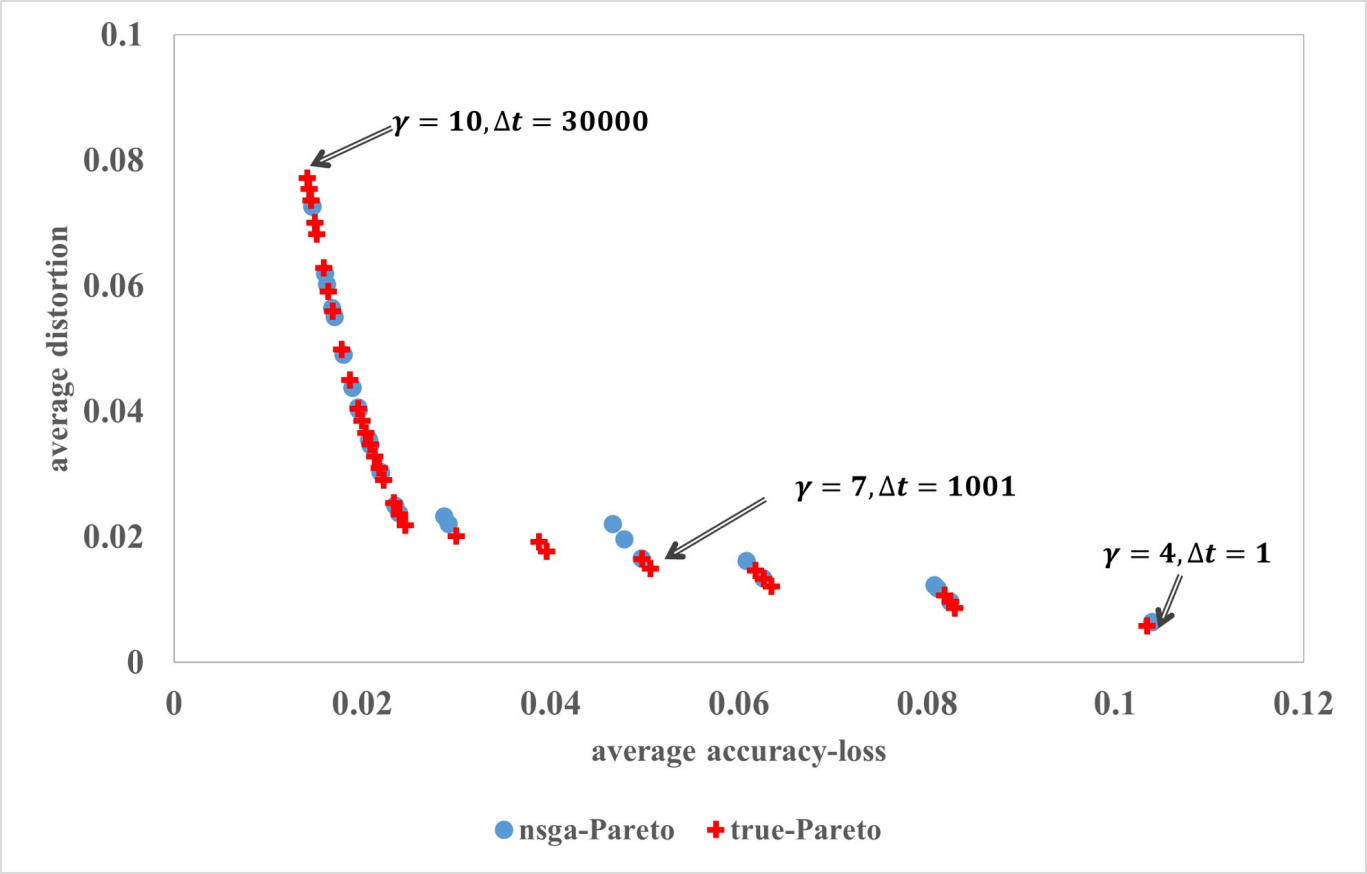
Pareto optimal front for optimization.

In the simulation, we choose *t*_0_ = 0, *t*_1_ = 20,000 and *t*_2_ = 50,000, with unit increase in indices to obtain the values of *j’*s. For simplicity, a unit increase in time coincides with the arrival of a reader. The media manager chooses the interval [*t*_0_, *t*_1_] over which the clicks received by the articles will be observed, and the observation corresponding to interval [*t*_1_, *t*_2_] is used to generate Pareto-optimal fronts. At *t*_2_, the simulation parameters, such as arrival rate of readers and average number articles read by them are updated using most recent previous observations corresponding to moving time-window of length *δt*. For the time interval [*t*_1_, *t*_2_] we present a selected set of 32 exemplar true optimal solutions, and solutions obtained through NSGAII [49] in Fig 6. The true Pareto-optimal solutions were generated using *non-dominated sorting* over the complete decision space.

In live implementation of Algorithm 2, for the selection of non-dominated solutions, an implementer may prefer to set a threshold for accuracy-loss and distortion metric to eliminate trivial solutions. For example, if threshold for accuracy-loss is 0.04 then all Pareto-front solutions having accuracy-loss less than 0.04 would be in the consideration set. Similarly, a threshold can also be set for the distortion metric. The pareto front allows media manager to examine all alternatives, and removal of bad alternatives before implementing any selected tuple (*γ*_*t*_, Δ*t*).

Importantly, the framework and mechanism presented in this section provides managers with the pareto front periodically as the news ecosystem dynamically evolves. In practice the manager will then have to periodically review the pareto front and choose the option (*γ*_*t*_, Δ*t*) that best represents the firm’s current interest in balancing conflicting objectives. It may be possible to treat this as an autonomous agent as well, constantly learning and adapting and automatically choosing a point on the pareto front, if the firm’s specific goals can be captured in greater detail. We note that the update process can also be implemented using multi-objective multi-armed bandits (MOMAB) [50]. However, we do not pursue MOMAB motivated dynamic adaption of FNRS in this research.

## Discussion

Viewing online news recommendation as a complex adaptive system, we proposed and studied a new model of feedback-based news recommenders that helped address the questions: (a) what kinds of unhealthy dynamics exist in commonly used top-N news recommendation? and (b) how can we design better algorithms to mitigate some of the undesirable outcomes?

To address the limitations of top-N recommendations, we present a novel recommendation technique based on a probabilistic feedback model. The feedback model can be considered a unifying framework for variety of known selection mechanism for recommendation. Through simulation we show that FNRS exhibits variety of self-organizing behavior and have been shown to be very effective in mitigating the undesirable emergent properties of top-N recommendation.

Further, we build upon the insights gained from the study of feedback models to develop a framework for dynamic adaptation of FNRS. We see dynamic adaptation of an FNRS as a combination of exploration and exploitation. Performing exploration and exploitation steps at regular interval requires selecting the appropriate level of feedback parameter based on two conflicting objectives: accuracy-loss and distortion. We develop these two metrics and their mathematical formulation in this paper extending the insights from metric M1 & M2, and discuss their role in feedback exponent selection. The optimization problem corresponding to accuracy-loss and distortion is categorized as "wicked problem" [19] due to, underlying randomness in objective functions and their conflicting nature, and subjectivity involved in selection of Pareto-optimal solutions. We use evolutionary algorithm NSGAII to generate non-dominated solutions of the multi-objective problem and present a method that aides FNRS to dynamically adapt with changing environment. The multi-objective evolutionary algorithm approach discussed in this research can be adapted to solve variety of wicked problems encountered in algorithm-dependent interconnected and interdependent systems.

## Conclusion

Due in part to the focus on “fake news” and the role those play in influencing opinion, there is substantial interest in the mechanisms through which people are influenced online. Our research in this paper on limitations of popular news recommender systems addresses a small but important piece of this bigger problem. By modeling news ecosystems as complex adaptive systems we have shown that specific unhealthy emergent behaviors can arise, but that these can be mitigated by a novel feedback-based model. We have further shown that media managers can use this framework to not just guide the emergent behavior of these complex systems, but to potentially optimize their objectives using a novel multi-objective framework. These are all new and important contributions in this paper.

From a practical perspective, this paper presents an effective mechanism for media managers to guide the self-organizing properties of the news ecosystem. Today current systems essentially use media editors to curate some of the available space and use automated systems such as top-N lists and personalized recommendations to populate other segments of the space available on their Web pages or apps. This does not offer much meaningful control over these algorithmic recommendations. Feedback models however, offer the potential for meaningful control, yet in a simple enough framework. Properties of FNRS as an autonomous agent can be embedded in design of dashboard for editors. Editors may track these dashboards to observe and set an appropriate decision parameters (for each recommendation list on their pages) as needed and observe the influence this brings into the highlighted articles.

Our recommendation framework studied here is easily generalizable to other contexts. The use of top-N lists extends beyond news articles alone. These are known to significantly influence mobile app downloads, product purchases and even access to scholarly articles (SSRN publishes top-N lists in several academic areas, for instance). In addition to “counts” of articles driving the feedback models it is possible to consider other metrics, such as similarity scores of collaborative filtering recommendations [51], as inputs to these functions in an obvious extension. In such cases, it is possible to generate product recommendation lists as well using feedback models within recommender systems. For example, Adamopoulos et al. [51] use probabilistic weighted sampling of *k* neighbors to develop a collaborative filtering algorithm and show that it outperforms popular recommendation approaches in terms of coverage, dispersion, prediction accuracy, and utility based ranking.

Though we discuss the use of FNRS from the implementers’ perspective. We believe, it also elegantly addresses some of the major concerns of the policy makers and the advertisers on the widespread use of top-N recommendation. Given that mere presence of an article or product in trending or top 10 list can influence the collective opinion of people and their consumption behavior [52]. There is growing recognition of need of better algorithms for fairness in data driven decision making [36, 53], manipulation of recommended lists [10, 54] and the less choice offered by top-N display lists [55]. In the context of top-10 app lists [55], notes that “*those who cannot get that visibility founder in obscurity*”. A further reason for count amplification in these top lists is the propagation of recommendations over social networks [56, 57], which has emerged as a major driver for internet traffic in recent years. Once an article (or app) makes such a list they are more likely to be picked up and propagated through social networks. Even if recommendation algorithms are designed with intent to project people’s preferences, they adjust what they do based on people’s behavior. And since they can reinforce human prejudices [58], by default, they are not *fair* and *just* in any meaningful way [59], and hence they may often generate socially undesirable results. The system influences readership or what succeeds by virtue of an artificial cutoff. It is such weaknesses that attract even manipulators to potentially game the system. The probabilistic FNRS presented here provides a framework that can also be used to elegantly address these challenges.

## Supporting information

S1 Appendix (DOCX)
