# Supplementary material for: A complex systems perspective of news recommender systems: Guiding emergent outcomes with feedback models
Source: PLoS One. 2021 Jan 7;16(1):e0245096. doi: 10.1371/journal.pone.0245096 (PMC7790545; doi:10.1371/journal.pone.0245096)
Supplement: S1 Appendix — (DOCX) [file pone.0245096.s001.docx]

**S1 Appendix**

**Details on simulation parameters**

For a few popular news websites, the number of unique visitors per day, is given in S1Table. The data on viewership for news websites was obtained from SimilarWeb (similarweb.com). Among other related information about a website, SimilarWeb also provides the information about page views per visitor.

We also had information about the number of stories generated per day for some of the popular news websites. For example, number of stories (URLs) generated per day at Huffington Post, Mail Online, and New York Times are approximately given by: 1600, 500-600, and 300-700 [1–3]. These observations were used to control for the news arrival process in the simulation, such that the ratio of arrival of readers to arrival of articles per day resembles a typical news website. For illustration, let’s assume that a news website has 60 million visitors per month, i.e. on average 2 million per day, and suppose 600 new articles are generated per day on this website. For simplicity, further suppose that new readers arrive sequentially and if a new article arrives then its arrival time coincides with arrival of a new reader. Then the arrival rate of articles w.r.t. arrival rate of readers would be $\rho=\left( \frac{600}{2,000,000} \right)= 0.0003$.

The estimates presented in S1 Table help us justify the selection of realistic parameters in the simulation (Table 2). To select the shape parameter $\lambda$, we use its average value reported in previous empirical findings on Web and mobile surfing [4,5].

| **S1 Table: Estimates of Number of Unique Visitors per Day for Media Sites** | | |
| --- | --- | --- |
| NEWS WEBSITES | ESTIMATED DESKTOP VISITORS  (in millions), OCTOBER 2014 | PAGE VIEWS PER VISITOR |
| BBC News (bbc.co.uk) | 404 | 3.63 |
| New York Times (nytimes.com) | 206 | 3.71 |
| Huffington post (huffingtonpost.com) | 273 | 2.00 |
| CNN (cnn.com) | 239 | 2.72 |
| Mail Online (Dailymail.co.uk) | 225.5 | 2.44 |
| The Guardian (Theguardian.com) | 168 | 2.10 |
| Fox News (foxnews.com) | 106 | 4.71 |
| The Telegraph (telegraph.co.uk) | 83.2 | 3.79 |
| The Times of India (timesofindia.indiatimes.com) | 77.2 | 3.13 |
| Forbes (forbes.com) | 69.1 | 4.17 |

**Reader behavior**

| **S1 Algorithm. A Reader’s Behavior** |
| --- |
| $p+ q+r=1;p, q, r$ are random numbers between 0 and 1.  *(p, q, r correspond to probabilities of reading from the popular list, a category, or breaking news on the “front page”. On category pages we retain p to be the probability of reading from the popular list, while (1-p) is the probability of continuing to select categories and read).*  view = 0  while (view <*L*)  Generate a random number $'rand'$ between 0 and 1  if front page  if (rand < p)  Select an article from most popular list randomly  Increase its count by 1  else if (rand > p && rand < p + q)  Select a category randomly  Select an article from the category according to read-index  Increase its count by 1  else  Select an article from Breaking News according to read-index  Increase its count by 1  else  if (rand < p)  Select an article from most popular list randomly  Increase its count by 1  else  Select a category randomly  Select an article from categorized list according to read-index  Increase its count by 1  view = view+1 |

**Sensitivity analysis for reading behavior**

We performed sensitivity analysis for different reading probabilities $\left( p,q, r \right)$ and these values are mentioned below and in Table-2.

| Case | $p,q, r$ |
| --- | --- |
| 1 | 0.3, 0.4, 0.3 |
| 2 | 0.3, 0.5, 0.2 |
| 3 | 0.4, 0.3, 0.3 |
| 4 | 0.4, 0.4, 0.2 |
| 5 | 0.5, 0.3, 0.2 |
| 6 | 0.6, 0.2, 0.2 |
| 7 | 0.7, 0.2, 0.1 |
| 8 | 0.8, 0.1, 0.1 |

We present the simulation results for selected parameters here. To the extent readers exhibit high to moderate preference for the recommended and recent articles, we observe the following trends.

The highest popularity amplification among the boundary articles happens in top-N recommendation due to significant drop in visibility of the (N+1)th article after the implementation of recommendation process ( see the trajectory of M1 corresponding to top-N in S4-S6 Figures). In the case of probabilistic recommendation, the popularity amplification trend between Nth and (N+1)th article is not deterministic because their initial click-count is almost same (see the trajectory of M1 corresponding to $\gamma=2, 4, 6, 8$ in S4-S6 Figures). Therefore, Nth and (N+1)th compete for the recommendation spot over the initial period of simulation. In the presence of positive feedback one among them eventually attains a significant lead over the other and gets selected for recommendation deterministically. We also find that the extent of popularity amplification between the Nth and (N+1)th article is controlled by feedback exponent.

Regarding M2 we find that (see S7-S9 Figures) the share of new click counts received by the articles which were not in the initial recommendation list depends upon: (a) the feedback exponent, and (b) the preference a reader exhibits for the recommended articles. For example, after sufficient time steps in S7 Figure approx. 70% of new Web traffic is received by the non-recommended articles, whereas in S8 Figure it is 40%. In the case of FNRS $\left( \gamma=2,4,6,8 \right)$ we observed that, as recommendation evolves over time a higher level of reinforcement is observed if readers exhibit higher preference for the recommended articles.

Finally, the results corresponding to Gini coefficient are reported in S1-S3 Figures.

We observed that readers exhibit moderate to high preference for the recommended articles the Gini coefficient exhibits increasing trend with respect to: (a) simulation time steps, (b) the extent of positive feedback, and (c) the preference for the recommended articles by readers. Among all recommendations used in the simulation top-N recommendation using sorting of click counts attains highest Gini value (see S1-S3 Figures). Therefore, we conclude that the use of recommendation is one of the major drivers of overall inequality in click counts of articles in the system.


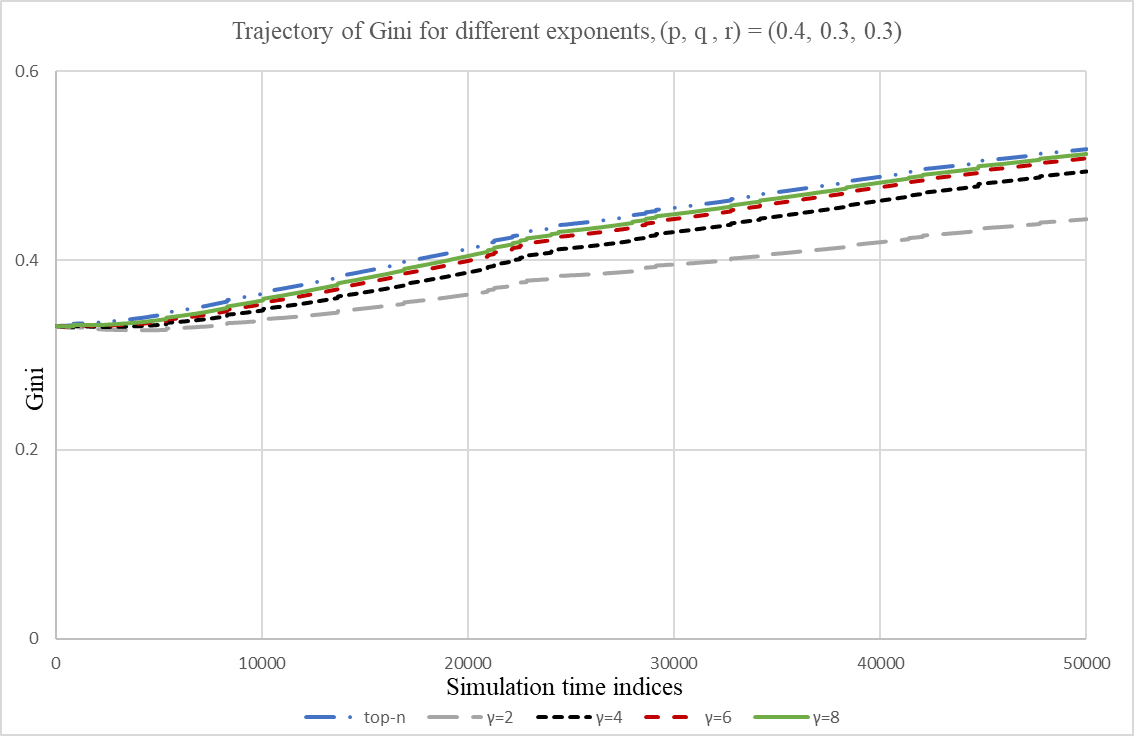

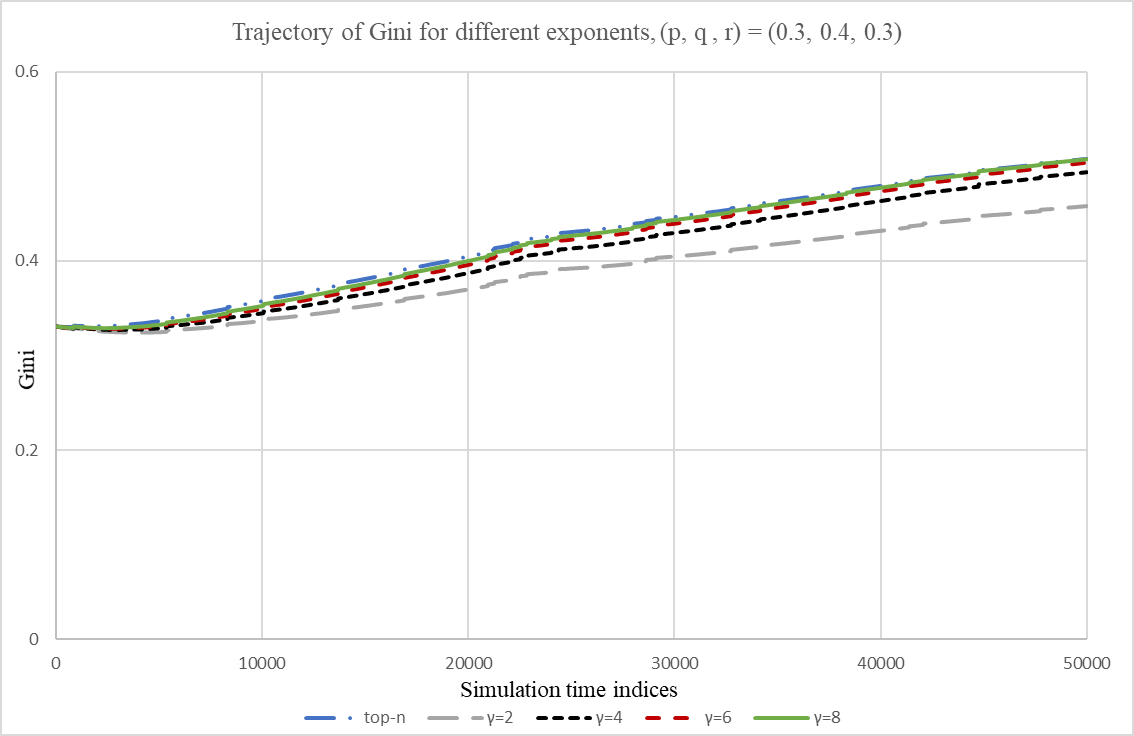


**S2 Fig. Inequality in popularity measured using Gini index (p, q, r) = (0.4, 0.3, 0.3)**

**S1 Fig. Inequality in popularity measured using Gini index (p, q, r) = (0.3, 0.4, 0.3)**


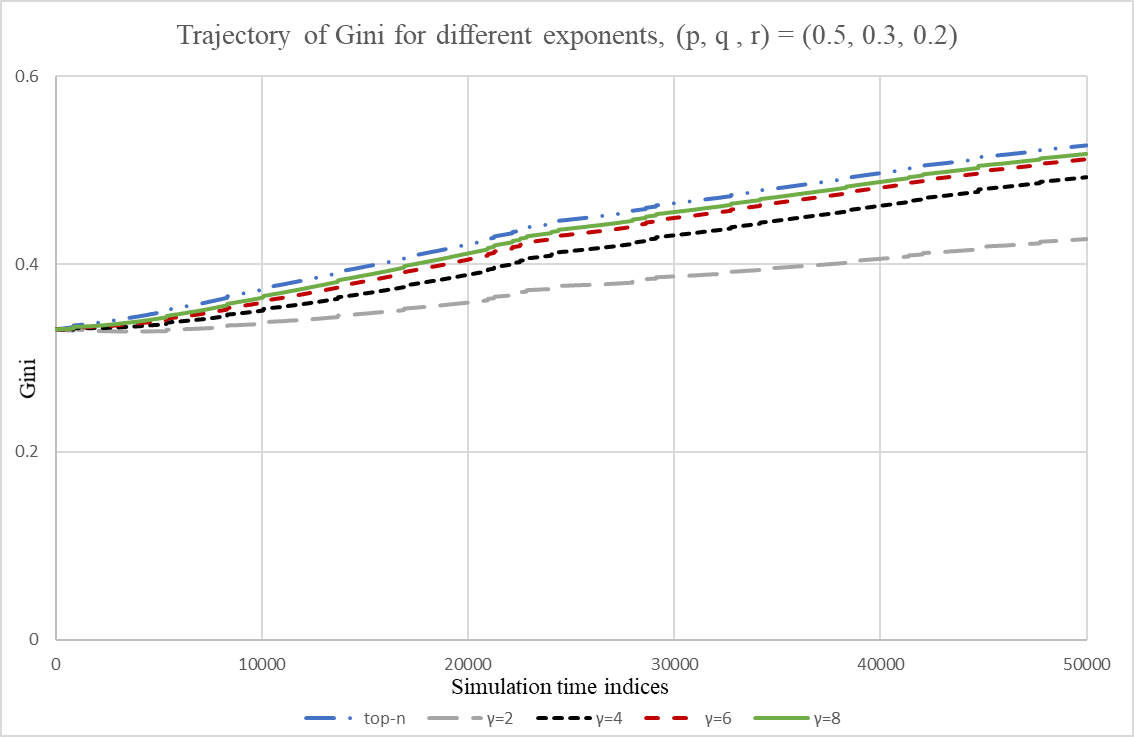


**S4 Fig. Boundary amplification of articles (p, q, r) = (0.3, 0.4, 0.3)**


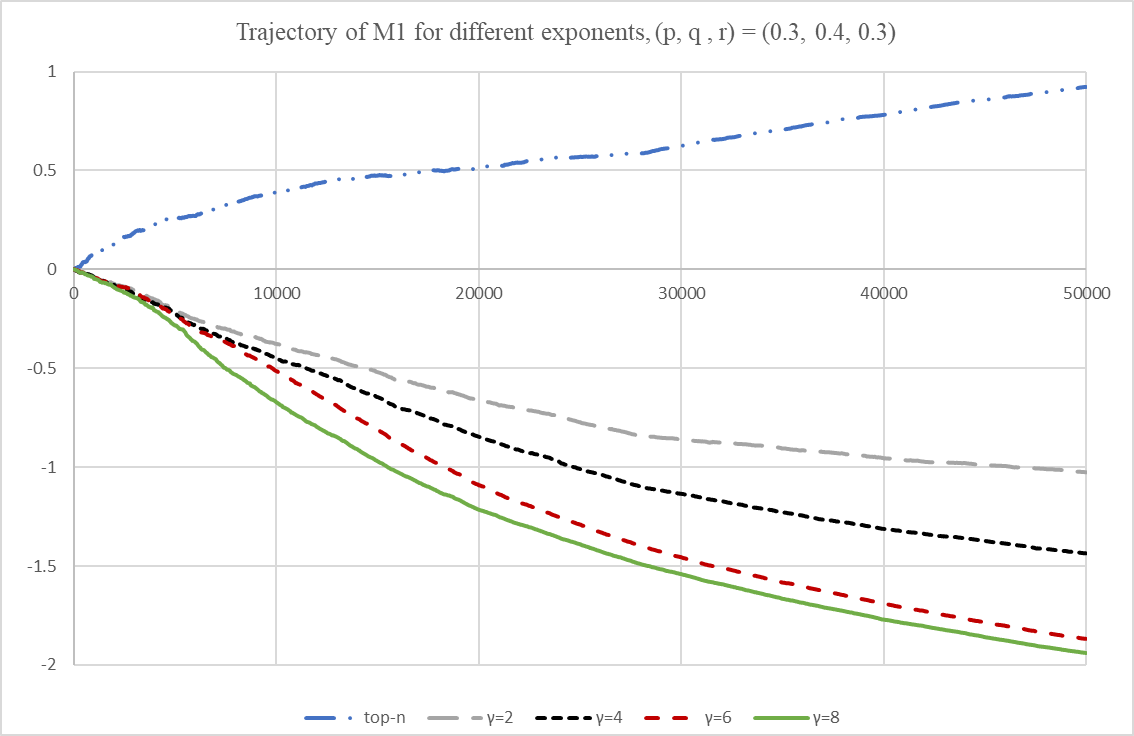


**S3 Fig. Inequality in popularity measured using Gini index (p, q, r) = (0.5, 0.3, 0.2)**

**S6 Fig. Boundary amplification of articles (p, q, r) = (0.5, 0.3, 0.2)**


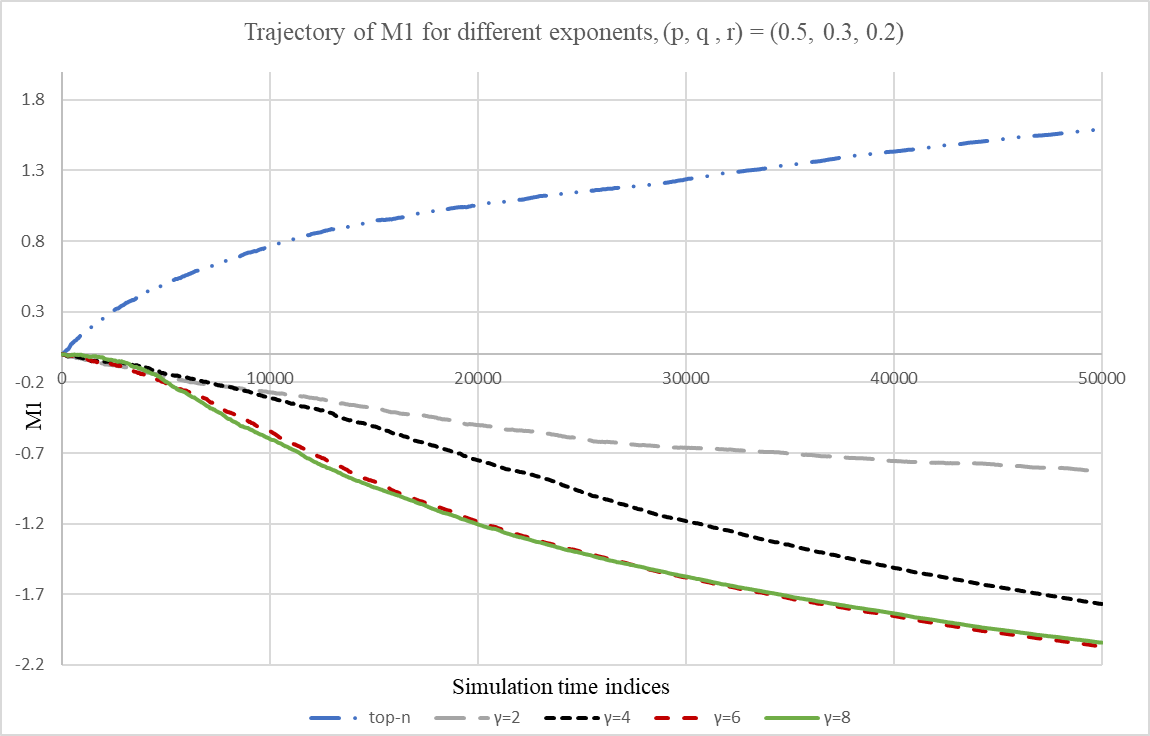


**S5 Fig. Boundary amplification of articles (p, q, r) = (0.4, 0.3, 0.3)**


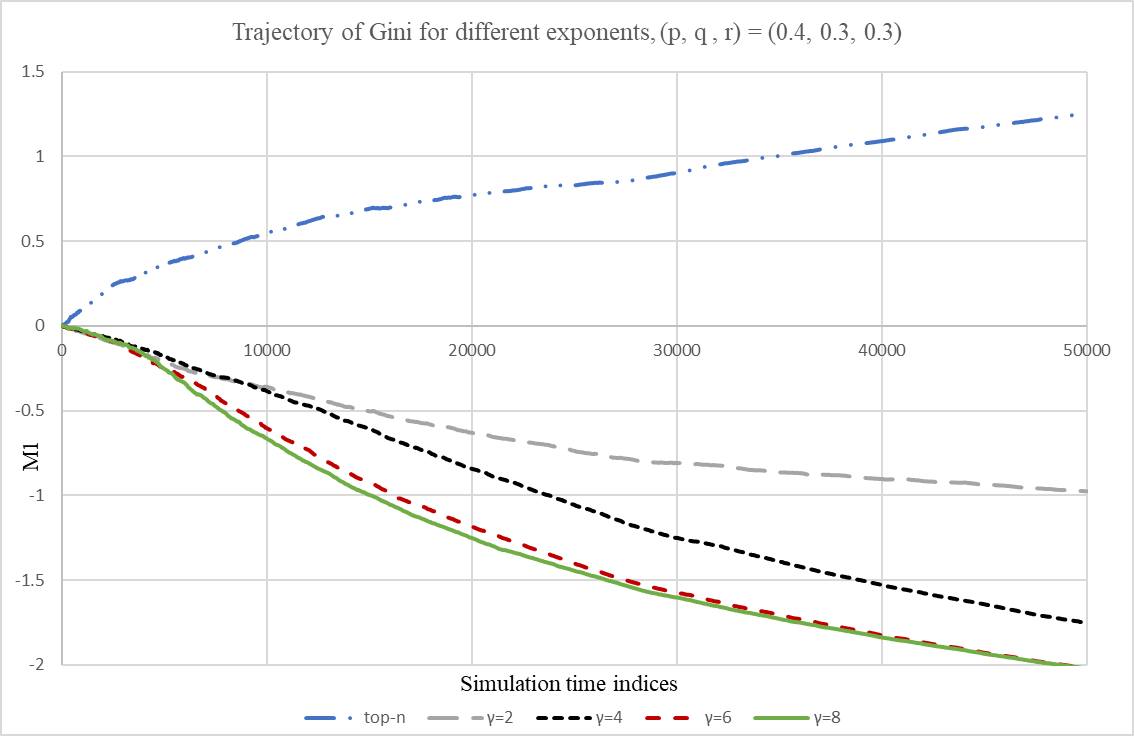


**S8 Fig. Reinforcement behavior of FNRS (p, q, r) = (0.4, 0.3, 0.3)**


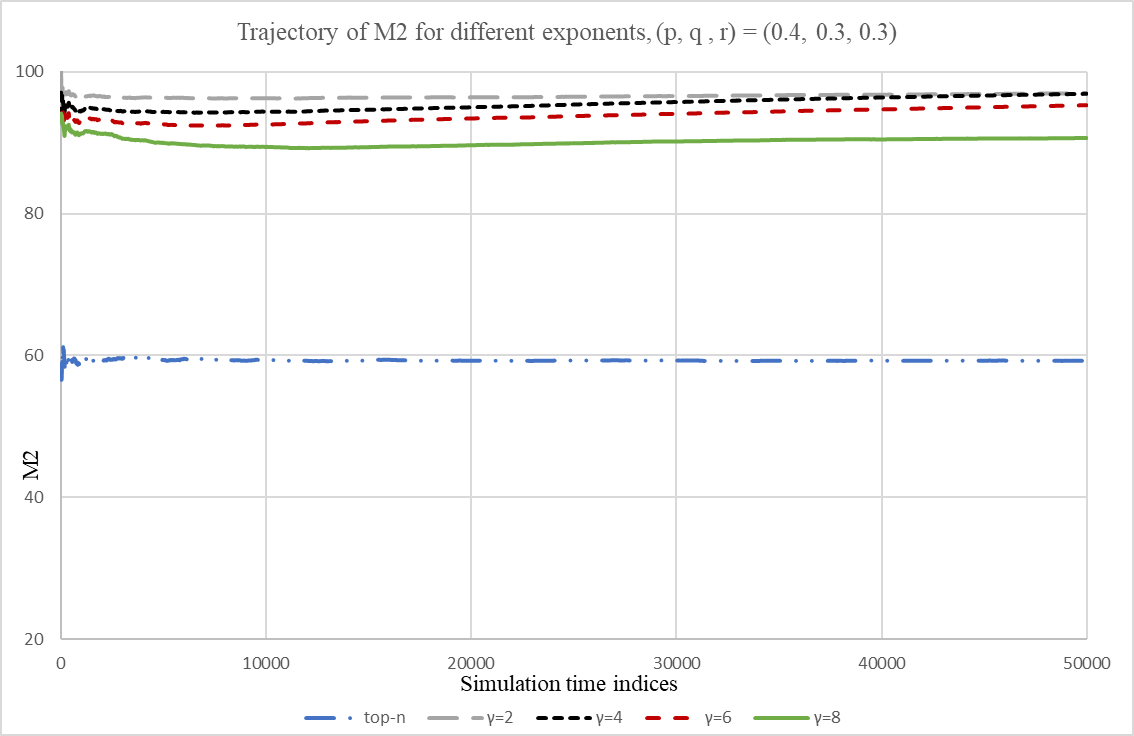


**S7 Fig. Reinforcement behavior of FNRS (p, q, r) = (0.3, 0.4, 0.3)**


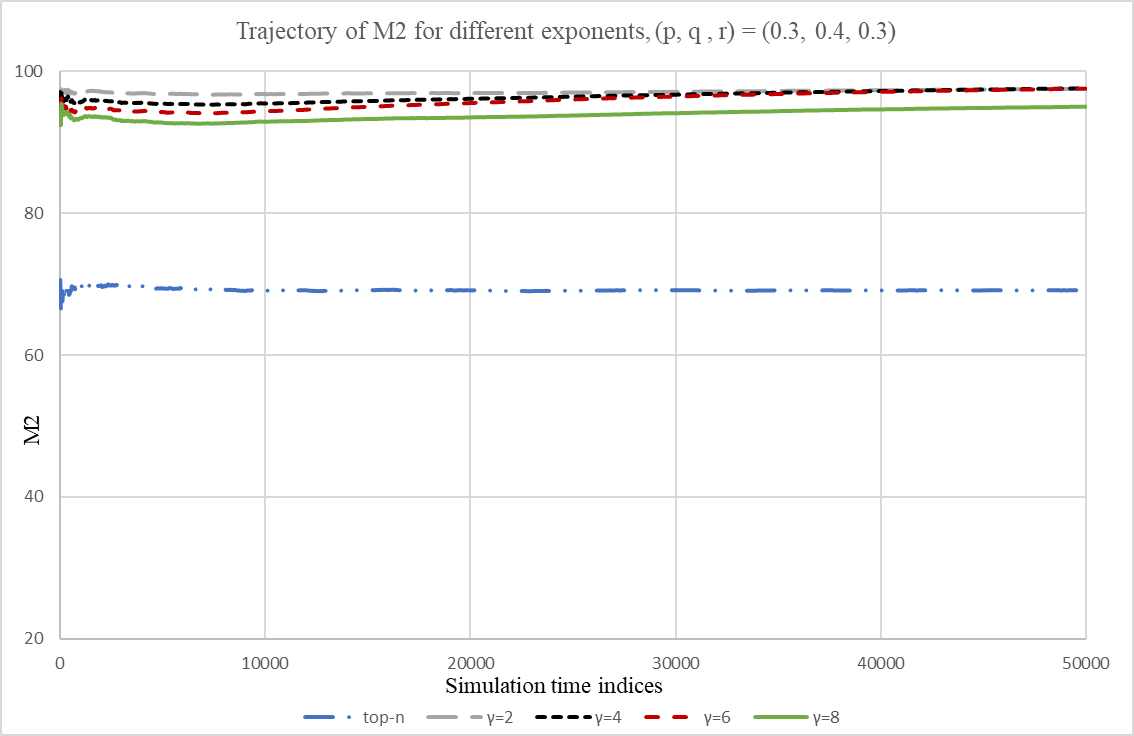


**S9 Fig. Reinforcement behavior of FNRS (p, q, r) = (0.5, 0.3, 0.2)**


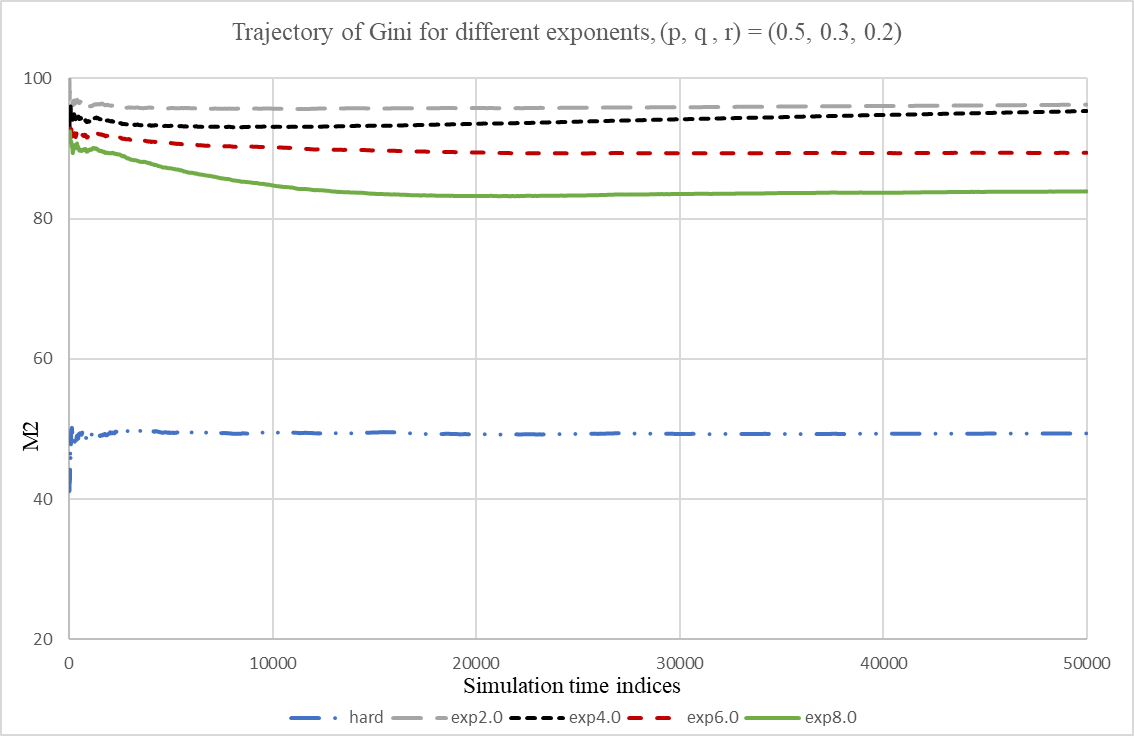


**Additional References (used in S1 Appendix)**

1. Usher B. Mail Online has grown ten-fold since its 2008 relaunch, but is it journalism? In: Press Gazette [Internet]. 2 May 2014. Available: https://www.pressgazette.co.uk/mail-online-has-grown-ten-fold-since-its-2008-relaunch-but-is-it-journalism/

2. Wills A. The Full New York Times Innovation Report | The New York Times | Journalism. [cited 10 Sep 2020]. Available: https://www.scribd.com/doc/224608514/The-Full-New-York-Times-Innovation-Report

3. Reid A. Can personalisation save the news homepage? | Media news. 20 May 2014. Available: https://www.journalism.co.uk/news/can-personalisation-save-the-news-homepage-/s2/a556786/

4. Halvey M, Keane MT, Smyth B. Mobile web surfing is the same as web surfing. Commun ACM. 2006;49: 76–81. doi:10.1145/1118178.1118179

5. Huberman BA, Pirolli PL, Pitkow JE, Lukose RM. Strong regularities in world wide web surfing. Science. 1998;280: 95–97.
